# A systematic review and meta-regression on international trends in the incidence of ulcerative colitis in children and adolescents associated with socioeconomic and geographic factors

**DOI:** 10.1007/s00431-024-05428-3

**Published:** 2024-01-17

**Authors:** Jens Weidner, Ivana Kern, Ines Reinecke, Franziska Bathelt, Ulf Manuwald, Elisa Henke, Michele Zoch, Ulrike Rothe, Joachim Kugler

**Affiliations:** 1 Institute for Medical Informatics and Biometry, Medical Faculty Carl Gustav Carus, TU Dresden, Fetscherstrasse 74, Dresden, 01307 Germany; 2 Department of Health Sciences/Public Health, Institute and Policlinic for Occupational and Social Medicine, Medical Faculty Carl Gustav Carus, TU Dresden, Fetscherstrasse 74, Dresden, 01307 Germany; 3 Thiem- Research GmbH, Carl-Thiem-Klinikum Thiemstr. 111, Cottbus, 03048 Germany; 4 University of Applied Sciences Dresden (FH-Dresden), Güntzstr. 1, Dresden, 01069 Germany; 5 GWT of TU Dresden, Dresden, Germany

**Keywords:** Epidemiology, Incidence, Ulcerative colitis, Inflammatory bowel disease, Pediatric

## Abstract

The incidence of ulcerative colitis (UC) among children and adolescents is rising globally, albeit with notable discrepancies across countries. This systematic review and meta-analysis aims to provide a comprehensive overview of the incidence rates of pediatric UC in various countries and explore potential influencing factors. A systematic literature search was conducted in PubMed and EMBASE (via OVID) for studies published between January 1, 1970, and December 31, 2019. Additionally, a manual search was performed to identify relevant systematic reviews. Meta-analyses and meta-regressions were employed to determine the overall incidence rate and examine potential factors that may influence it. A total of 66 studies were included in the qualitative analysis, while 65 studies were included in the meta-analysis and 50 studies were meta-regression. The study reports a rising incidence of pediatric UC in several countries but significant differences across geographic regions, with no discernible global temporal trend. In addition, our meta-regression analysis showed that geographic location and socioeconomic factors significantly influenced the incidence of UC.

*Conclusion*: Our findings indicate a rising incidence of pediatric UC in numerous countries since 1970, but with significant geographical variation, potentially presenting challenges for respective healthcare systems. We have identified geographic and socioeconomic factors that contribute to the observed heterogeneity in incidence rates. These findings provide a foundation for future research and health policies, aiming to tackle the growing burden of UC among children and adolescents.

**Table:** 

**What is Known:** *• The incidence of ulcerative colitis in childhood and adolescence appears to be increasing worldwide and varies internationally.* *• Environmental and lifestyle factors are suspected as potential causes.*	
**What is New:** *• Our results highlight that the heterogeneity in incidence rates can be attributed to geographic and socio-economic factors.*

**Supplementary Information:**

The online version contains supplementary material available at 10.1007/s00431-024-05428-3.

## Introduction

Ulcerative colitis (UC) is an immune-modulated disease and, together with Crohn’s disease (CD) and Colitis indeterminata (CI), classified as an inflammatory bowel disease (IBD). Clinically, UC is characterized by inflammation confined to the colon and rectum, resulting in diffuse friability and erosions to the mucosa and submucosa of the colon, and is associated with bleeding. Typically, the disease initiates in the rectum and then extends continuously in a proximal direction [1, 2]. IBD spectrum diseases are often diagnosed in childhood and adolescence. Approximately 20% of IBD cases are diagnosed before the age of 20, with an adverse shift in the age of diagnosis to the early childhood years [3–6]. For instance, in the USA, 2–4 cases of UC per 100,000 children (aged 10–19 years) are reported [7]. UC is a worldwide chronic illness, with the most noteworthy rates of occurrence recorded in North America and Northern Europe. Recently, countries from Asia and South America that were not previously associated with high UC incidence rates have reported increasing rates of new cases [3, 8, 9]. However, international epidemiologic data for UC vary considerably by geographic region and trends over time [10, 11]. Therefore, the escalating incidence of UC is poised to present significant challenges to the healthcare systems of respective countries in the forthcoming years [5, 12]. The etiology of UC has not been conclusively determined. However, a multifactorial interaction of genetic, environmental, and liver factors, as well as dysregulation of the mucosal immune system, is thought to influence the pathogenesis of UC [1, 13, 14]. Particularly, the impact of the Western lifestyle on immune-modulated diseases, such as UC and CD, is a subject of discussion. These diseases exhibit correlations not only with socioeconomic factors but also with geographic and environmental variables [6, 15–17]. The clinical course of children with UC differs from adults, so the direct health care costs may be higher in children with UC compared with adults. However, the direct burden of disease is also not insignificant for affected children: The disease is associated with growth retardation, increased risk of osteoporosis, and psychosocial problems [15, 18–20]. The treatment of UC in children and adolescents holds considerable medical and health economic importance, given the increasing prevalence of pediatric cases of IBD and the disease’s tendency to manifest at an earlier age, as supported by numerous international studies [10, 15, 20, 21]. It is essential to establish epidemiological associations to inform future research and public health policies that aim to tackle the growing societal and individual burden of disease of IBD spectrum disorders in young people. Literature that quantifies the influence of factors that explain the increasing incidence of UC and IBD is limited. The objective of this study is to investigate global trends in the incidence of UC since 1970, aiming to identify and quantify the factors contributing to the observed increase in incidence.

## Methods

We conducted a systematic literature search on the spectrum of IBD diseases in childhood and adolescence. Our search included terms such as Crohn’s disease, ulcerative colitis, and clinical examination as well as other keywords such as incidence, prevalence, children, adolescents, and pediatrics, which we logically linked with Boolean operators. Since patients were not directly involved in this study, an ethics vote was not required for the systematic literature review. The systematic review search has been registered in Prospero (PROSPERO-NR: CRD42020168644). The guidelines outlined in the PRISMA statement were followed for the systematic review [23]. The systematic literature search was conducted using the PubMed and EMBASE databases via OVID. Additionally, a manual search of the bibliographies of previously published systematic reviews was conducted. We refer to the published study protocol for more details on the methodology [22]. For the present study, a previous systematic literature search in 2021/2022 was updated to include studies published through 2021 and include literature from 1970 to 2019. The title-abstract and full-text screening, as well as the data extraction and consistency checks, were done by two authors independently. In case of disagreement, a third person was consulted for mediation. Quality assessment of all included studies was done using critical appraisal instruments CASP [24] and SIGN [25]. Additionally, we performed a risk of bias analysis following the procedure outlined in the Cochrane Handbook [26] (see Risk of Bias in Supplementary Material). We did not exclude studies of poor quality from the quantitative synthesis to avoid losing information. 

### Data extraction

**Fig. 1:**
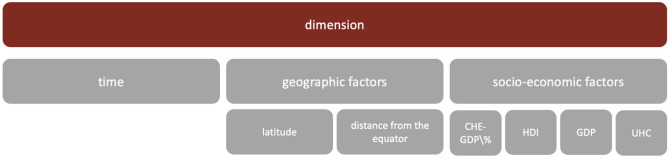
Dimensions of the factors for the meta-regressions

All included studies were reviewed for incidence rates and study characteristics using a standardized tabular summary of results. In case of missing data, the authors were contacted to obtain the necessary information. Subsequently, the data were exported to a database and prepared for statistical analysis. For studies by a single author that reported multiple incidence rates for children and adolescents, mean values of incidence rates and study sizes were calculated for each observation period. In preparation for meta-regression, potential moderators of heterogeneity were categorized along three dimensions: time, geographic factors, and socioeconomic factors (Fig. 1). Geographic data was obtained by extracting information from Geoplaner V.3.1 [27]. In the case of studies conducted for nationwide or multicentered studies, the mean latitude for the respective country or area was utilized. Additionally, the mean distance to the equator was computed based on the mean latitude. To investigate the factors of the socioeconomic factors (3rd dimension), the percentage of gross domestic product allocated to health (CHE-GDP/%) was utilized, which was extracted from the “Health expenditure and financing” database of the Organization for Economic Co-operation and Development (OECD) [28]. The Human Development Index (HDI) was also included as socioeconomic factor. The HDI evaluates a country’s level of development by combining life expectancy at birth, expected years of schooling, and gross national income per capita [29]. HDI value was taken from the United Nations Development Programme’s Human Development Reports [29], which were averaged for statistical analysis starting from 1990. Additionally, the gross domestic product (GDP) of the included countries was used from the Genesis database of the Federal Statistical Office for socioeconomic factor analysis [30]. Furthermore, the Universal Health Coverage (UHC) service coverage index SDG 3.8.1 was extracted from the WHO database. The UHC index quantifies the coverage of essential health services and is defined as the average coverage based on tracer interventions that include reproductive, maternal, newborn, and child health, infectious diseases, noncommunicable diseases, and service capacity and access among the population [31].

### Statistical analysis

Random-effects meta-analyses and meta-regressions were conducted to assess the variability of incidence rates, employing R software version 4.2.2 and the Metafor package version 3.8-1 [32]. The meta-analysis was performed on a logarithmic scale (log incidence rates) utilizing the general inverse variance method. This approach allows the transformation of the incidence rates into a symmetric distribution, which is better suited for statistical testing and estimation. The use of a logarithmic scale enables the combination of studies with differing sample sizes and time periods [33–35]. To estimate random effects and the extent of heterogeneity, we utilized the restricted maximum likelihood estimator (REML). To identify further moderators of heterogeneity in incidence rates, we constructed a multivariate model for the meta-regression. The dependent variable was the pooled incidence rates for the observation period, with the starting time of each study averaged and assigned. The mean distance of the included countries from the equator, HDI, CHE-GDP/%, GDP, and UHC were included as additional independent variables in the regression model. As data for moderators from the socioeconomic dimension are only available from the year 1990 onwards, not all identified studies could be considered for the meta-regressions. Therefore, only 50 out of the total 66 studies were included in the moderator analyses.

In addition to the estimate of ${\tau }^{2}$, we reported the *Q*-test for heterogeneity and the ${I}^{2}$ statistic based on the categories provided by Higgins et al. [32, 36]. The influence of the moderators was evaluated using the ${R}^{2}$ statistic as a measure of the explained heterogeneity [36]. We set an a priori significance level of 5% for all statistical methods.

## Results

### Data basis and general assessment of studies

This study covers a 50-year observation period with studies on UC from 1970 to 2019. In 2019, the initial search identified a total of 3153 studies. A search update identified another 83 records, with 77 of them undergoing screening, ultimately resulting in the inclusion of 5 studies eligible for synthesis. In total, the search yielded 81 findings from 29 different countries, encompassing data related to CD, UC, and CI. In this study, 65 studies focusing on ulcerative colitis were included in the qualitative synthesis. For the purposes of meta-analysis, we utilized 65 eligible studies from 24 different countries, while 50 eligible studies were employed for meta-regression (Fig. S1 in Supplementary Material).

Out of the 65 studies, the incidence rates on the linear scale ranged from .02/10^5^ to 13.77/10^5^. Upon conducting a random-effects meta-analysis, the mean incidence rate was calculated to be 1.78/10^5^ (95% CI, 1.35/10^5^–2.34/10^5^), which corresponds to −10.93 on the log scale (with a range of −15.42 to −8.89) (Fig. S2 in Supplementary Material). However, the high ${I}^{2}$ value of 97.34% indicates that the significant result variation cannot be solely attributed to sampling differences but helps to pinpoint possible factors to explain those variations in study outcomes. Surprisingly, we found that the between-study variance had a much greater impact on the individual weights assigned to each study, compared to the study-specific variance (i.e., the sampling effect), which had a relatively minor influence. As a result, in the random-effects meta-analysis, the studies have similar relative weights.

### Time as a moderator of UC incidence

**Fig. 2:**
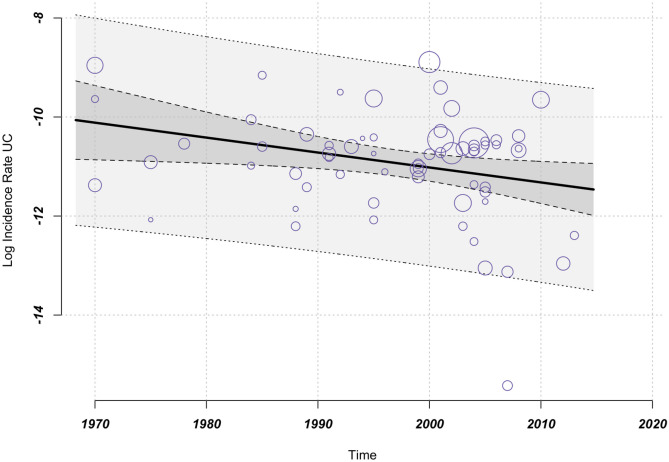
Meta-regression: time as moderator, ME model (*k* = 65, estimator: REML): test of moderators *F*-test = 5.25 *P* = .02, *I*^2^ = 97.07%, variance explanation via *R*^2^ = 7.06%

A meta-regression analysis was performed, with treating time as a continuous independent variable and UC incidence rates as the dependent variable. Figure 2 indicates that the moderator of time has a statistically significant effect on the incidence rates of UC; however, the incidence rates exhibit an unexpected decrease. Specifically, the multiplicative change factor is 0.97. Despite a statistically significant test of moderators (*P* = .02), the moderator of time accounts for only 7% of the observed heterogeneity, which suggests that a considerable proportion of the heterogeneity remains unexplained (test of moderators *P* = .02, ${I}^{2}$ = 97.07%, ${R}^{2}$ = 7.06%) (Fig. 2). The results indicate the existence of other moderators examined to help explain the observed heterogeneity. A negative trend in UC incidence rates accompanied by an increase in heterogeneity is shown. The impression of greater geographic dispersion is reinforced by the presence of studies reporting low incidence values, particularly from South America and Asia.

### Geographical factors as moderators of UC incidence rates

**Fig. 3:**
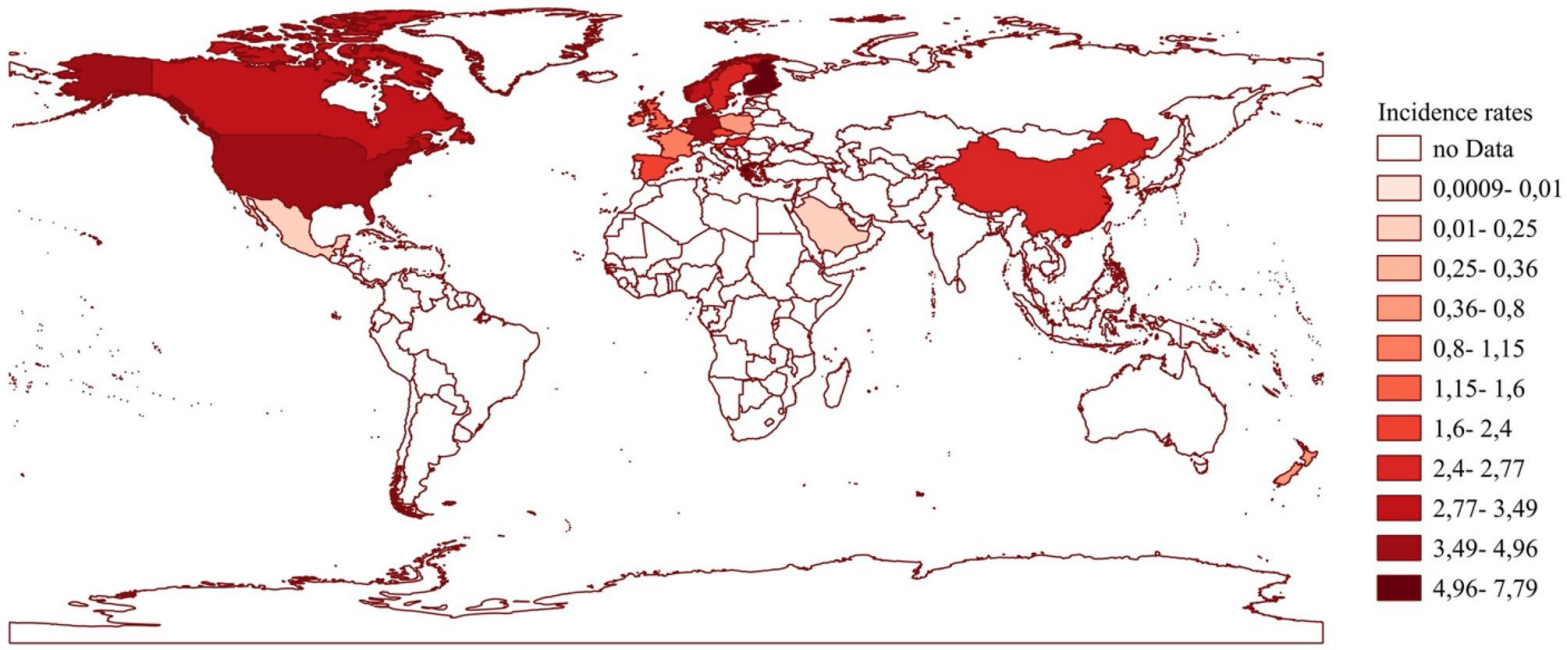
Geographical distribution of ulcerative colitis incidence (raw data)

The second-highest mean incidence rate per 100,000 children and adolescents was observed in Greece (7.50 new cases/10^5^) after Finland (7.79/10^5^). The USA showed the third-highest incidence rate of UC at 4.95/10^5^ followed by Germany (4.49/10^5^) and Canada (3.49/10^5^). The lowest incidence rates were reported in studies from Mexico (0.02/10^5^) and Asian countries (Saudi Arabia 0.19/10^5^, Taiwan 0.22/10^5^), although these geographies are underrepresented regions in terms of the number of included studies (Fig. 3). However, it was noticeable that China, with an incidence of 2.58/10^5^, differed from other Asian countries in the level of incidence rate. These data suggest geographic heterogeneity, which we consider further at the continental level.

A meta-regression analysis including the continents variable explained 60.4% of the heterogeneity and indicated a significant test for moderators (*P* $<$ .001). To examine the potential moderating effect of time, we compared this model with a more complex model that included time as a factor. However, our analysis showed no significant improvement in ANOVA, indicating the moderator was masked by other factors.

In a subsequent analysis, we examined the progression of ulcerative colitis incidence over time for each continent. Our results suggest that the incidence of UC has developed differently across continents, with no significant outcomes from the meta-regression analysis. Therefore, we cannot draw robust conclusions about trends in the incidence of UC (Table 1, Fig. S10 in Supplementary Material).

**Table 1:** Meta-regression results

Continent	Estimate	se	*z*-value	*P*-value	95% CI	*I*^2^	*R*^2^	Exp. Coef
Asia	−.057	.11	−.49	0.62	−.285 to .170	88.9%	.00%	.9441
Australia/Pacific^a^	.338	.28	1.19	0.23	−.21 to .89	-	-	1.402
Europe	.006	.012	.49	0.62	−.018 to .031	90.0%	.00%	1.006
North America	−.011	.018	−.59	0.59	−.047 to .025	97.4%	.00%	1.030
South America^a^	-	-	-	-	-	-	-	-

Dependent variable incidences UC, independent variables: Time

^a^Too few observations for regression analysis, a multiplicative change factor $>$ 1 increasing IR, $<$ 1 decreasing IR

**Fig. 4:**
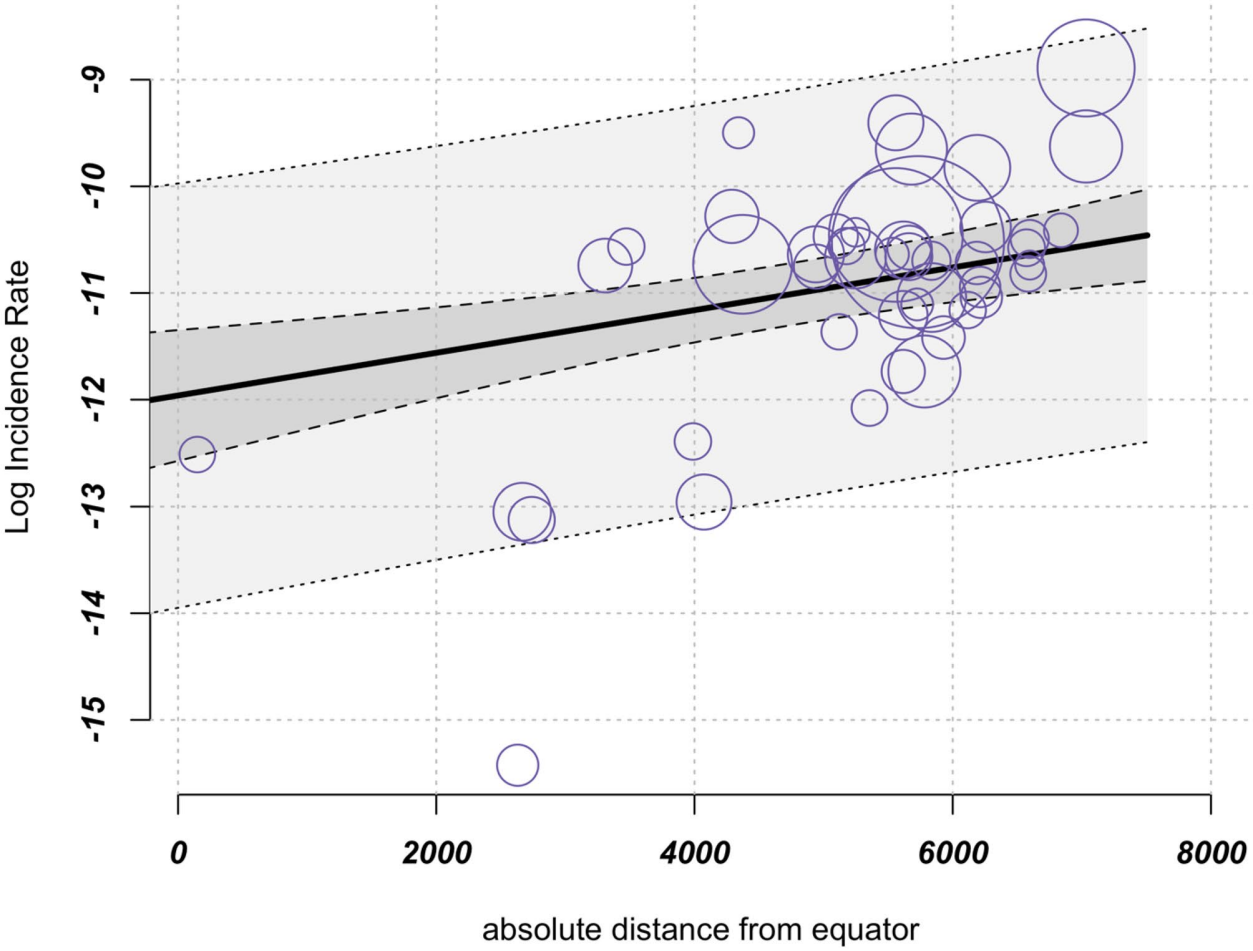
Meta-regression: increasing incidence with increasing distance from the equator; (*k* = 50, estimator: REML): test of moderators *F*-test = 11.81 *P* < .001, *I*^2^ = 97.31%, variance explanation via *R*^2^ = 20.76%

As the results reveal geographic heterogeneity in incidence rates, we postulated that latitude or the average distance from the equator could be influencing UC incidence. To investigate this possibility, a meta-regression analysis was conducted with absolute distance from the equator as a moderator. The findings indicate a significant positive relationship between UC incidence and increasing distance from the equator (Fig. 4). When we extrapolated the results to a distance of 1000 km, we observed a 0.2% increase in the incidence rate of UC. Our test for moderators produced a significant result (*P* < .001). However, considering the significant heterogeneity in the study outcomes, the distance from the equator only moderately contributed to better explain the variance (*R*^2^ = 20.76%).

## Socioeconomic factors as moderators of UC incidence rates

**Fig. 5:**
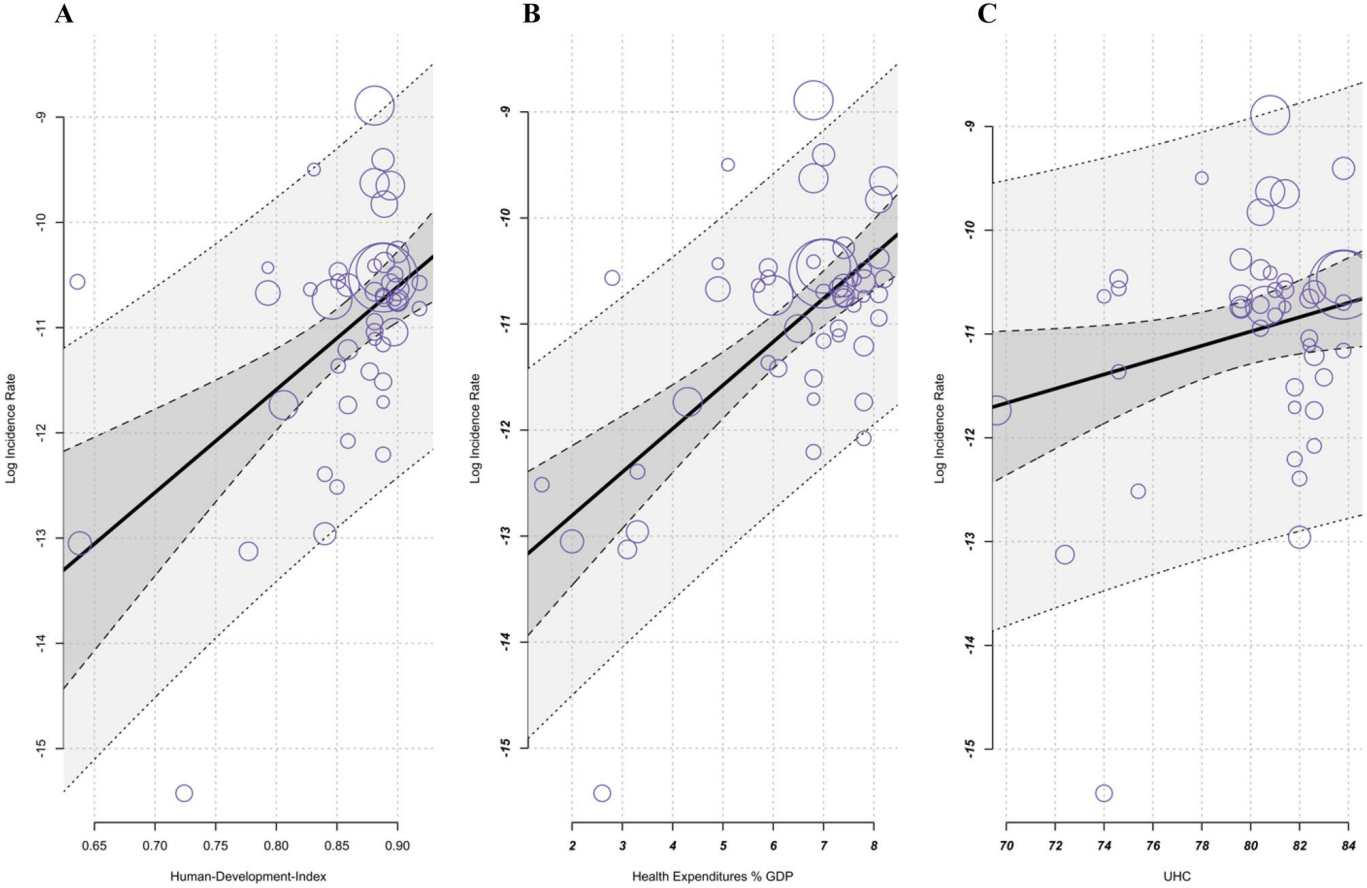
Meta-regression. **A** Increasing incidence with increasing Human Development Index (HDI); (*k* = 50, estimator: REML): test of moderators *F*-test = 16.83 *P* < .0001, *I*^2^ = 96.94%, variance explanation via *R*^2^ = 29.73%. **B** Increasing incidence with increasing health expenditure (CHE-GDP%); (*k* = 50, estimator: REML): test of moderators *F*-test = 33.28 *P* < .0001, *I*^2^ = 96.1%, variance explanation via *R*^2^= 45.85%. **C** Increasing incidence with increasing UHC service coverage index SDG 3.8.1 (UHC); (*k* = 50, estimator: REML): test of moderators *F*-test = 4.19, *P < *.001, *I*^2^= 97.56%, variance explanation via *R*^2^= 8.56%

The results of the associated meta-regression analysis of socioeconomic factors showed that HDI, CHE-GDP/%, and UHC index acted as moderators. Consequently, the incidence of UC showed an upward trend when the values of each moderator increased. Remarkably, the moderators’ health expenditure, accounting for approximately 46% of GDP, and HDI, around 30%, accounted for a significant portion of the heterogeneity (see Table 2 and Fig. 5). To avoid complications arising from collinearity and the potential for unreliable coefficient estimates resulting from intercorrelations among socioeconomic factors, we decided against a combined regression model.

**Table 2:** Meta-regression results

Moderator	Estimate	SE	*z*-value	*p*-value	95% CI	*I*^2^	*R*^2^
HDI	9.77	2.38	4.31	< .0001	5.11–14.44	96.94%	29.73%
CHE-GDP/%	.41	.07	5.77	< .0001	.27–.55	96.10%	45.85%
UHC	.07	.03	2.05	.04	.003–0.14	97.56%	8.56%

Dependent variable incidences UC, independent variables: HDI, CHE-GDP/%, UHC

ME Model (*k* = 50, estimator: REML)

## Discussion

The systematic review including meta-analysis and meta-regression investigated global trends in the incidence of UC. While several individual studies show an increase in incidence rates for UC, the available evidence to support and quantify this increase appears to be of limited quality (see Risk of Bias Analysis in Supplementary Material). Different study designs have complicated the comparison of incidence rates over time, which could further contribute to the significant heterogeneity observed in these rates. For our present systematic review, we thoroughly evaluated 65 studies from 24 countries, spanning a 50-year observation period. Our analysis revealed substantial heterogeneity in UC incidence rates, which was confirmed through a meta-analysis using a random-effects model (Cochrane *Q* = 1751.04, *P < *.0001, *I*^2^ = 97.34%).

Regardless of the substantial heterogeneity present in the data, our study yielded several noteworthy findings. Firstly, we found no conclusive evidence supporting a consistent global trend of increasing incidence rates for UC over time. Although specific countries may exhibit an increasing trend in incidence rates, the inclusion of studies from a broader range of countries has resulted in an amplification of between-study heterogeneity. Secondly, we observed a recognizable association between geographic location and UC incidence rates, with higher incidence rates for countries located further away from the equator. This suggests a potential influence of factors correlated with latitude on the occurrence of UC. Thirdly, we identified a similar pattern with regard to socioeconomic factors, whereby higher scores on socioeconomic indicators were significantly correlated with higher incidence rates of UC. This highlights a potential association between socioeconomic status and the occurrence of the disease.

Regarding the geographic variations in UC incidence rates, multiple studies have reported a north-south gradient. For instance, Nerich et al. (2006) examined the impact of latitude on the geographic distribution of UC [37]. Similarly, Weidner et al. (2023) reported a significant association from a global perspective for CD [6]. Considering recent epidemiological studies reporting increasing incidence of inflammatory bowel diseases worldwide, including southern countries and particularly the southern hemisphere [9, 38, 39], we thus opted to incorporate the absolute distance from the equator as a factor in our study. This decision was made to elucidate and quantify the relationship between incidence and geographical location. The result of our meta-regression showed that incidence rates increased with increasing distance from the equator. Our findings on the socioeconomic factors contribute to the hypothesis that UC correlate with industrialized, urbanized societies, largely attributable to a Western lifestyle and other associated environmental factors [6, 15] associated with higher socioeconomic values. It is also known that the incidence and prevalence of UC, as with CD, vary among countries with different HDI levels [6, 40, 41]. While there are only a few epidemiological studies on UC in underdeveloped and developing countries, the incidence of UC seems to be increasing worldwide, even affecting countries that were previously considered low-risk [38, 42]. The incidence and prevalence have also been observed to increase among children and adolescents in developing countries, attributed to rapid modernization and Westernization of the population [15]. In this regard, we also align with the views of Takahashi et al. (2018) and Ananthakrishnan et al. (2015) that the level of development of countries and the Western lifestyle are associated with the incidence rate [38, 41]. However, our study does not establish causality, and further investigations are required for this purpose. Lately, observational research on real world data (RWD) gains importance and research networks are established worldwide, such as the Observational Health Data Sciences and Informatics (OHDSI) community to foster studies on large scale based on the Observational Medical Outcomes Partnership (OMOP) Common Data Model (CDM). Thus, observational studies on RWD would be beneficial to investigate this topic in the future.

### Strengths and limitations

The study benefits from a substantial temporal scope (50 years), which allows for a comprehensive examination of the topic over time. In addition, many studies were included in the analysis, which increases the power of the results. The use of meta-analysis methods facilitated the integration of data from multiple studies, allowing for a more comprehensive examination of the research question. The systematic literature search is limited to two electronic databases and thus may result on missing studies not available electronically or indexed in other databases. In addition, the study applied a language restriction that excluded publications that were not written in English, Spanish, French, or German. We controlled the risk of publication bias using the Eggers regression test, rank correlation test, trim and fill analysis, and fail-safe N analysis (Rosenberg method). Although these methods did not statistically indicate a bias due to publication bias, a small bias cannot be completely ruled out.

## Conclusion

The present study did not reveal a consistent global temporal trend indicating an increase in UC incidence rates. However, individual countries or regions may exhibit such trends. Notably, our findings highlight that a significant portion of the heterogeneity observed among the published study results can be attributed to geographic location and socioeconomic factors. Consequently, our study provides quantitative estimations of these trends specific to UC in childhood and adolescence. Nevertheless, in order to establish causal relationships and better understand potential risk factors, further investigations are required, particularly encompassing countries with lower levels of development. To facilitate these endeavors, the adoption of internationally standardized and interoperable registries, alongside the provision of health data through federated networks based on a CDM, such as the OMOP CDM, would be advantageous. OMOP CDM aligns most closely with the criteria for facilitating data sharing in longitudinal studies [43]. These registries and data networks would facilitate the comprehensive and comparable collection and sharing of data, thereby enhancing our understanding of UC and promoting evidence-based strategies for prevention and intervention.

### Supplementary information

Below is the link to the electronic supplementary material.

Supplementary file1 (PDF 50 KB)

Supplementary file2 (PDF 57 KB)

Supplementary file3 (DOCX 2260 KB)

## Data Availability

The data sets generated and/or analyzed during this study are available from the corresponding author upon reasonable request.

## Abbreviations

abs.Dis: Absolute distance from equator
CD: Crohn’s disease
CDM: Common Data Model
CI: Colitis indeterminata
CHE-GDP/%: Health expenditure as a percentage of GDP
GDP: Gross domestic product
HDI: Human Development Index
IBD: Inflammatory bowel disease
OHDSI: Observational Health Data Sciences and Informatics
OMOP: Observational Medical Outcomes Partnership
REML: Restricted maximum likelihood
UC: Ulcerative colitis
UHC: UHC service coverage index SDG 3.8.1
