# Supplementary material for: A systematic review and meta-regression on international trends in the incidence of ulcerative colitis in children and adolescents associated with socioeconomic and geographic factors
Source: Eur J Pediatr. 2024 Jan 17;183(4):1723–32. doi: 10.1007/s00431-024-05428-3 (PMC11001685; doi:10.1007/s00431-024-05428-3)
Supplement: Supplementary file 1 — Supplementary file1 (PDF 50 KB) [file 431_2024_5428_MOESM1_ESM.pdf]

Author	Year	Recruitment procedere & follow up	Outcome definition und measurement	Outcome source and validation	Confounding and effect modification	Analysis method: methods to reduce research specific bias	Funding	Conflikt of Interesst
Abramson et al.	2010	🟢	🟢	🟢	🟢	🟢	🟢	🟢
Adamiak et al	2013	🟢	🟢	🟢	🟢	🟢	🟢	🟢
Ahmed et al.	2006	🟢	🟢	🟢	🟡	🟡	🟢	🟢
Armitage et al.	2001	🟢	🟢	🟢	🟡	🟡	🟢	🟢
Ashton et al.	2014	🟢	🟢	🔴	🔴	🟡	🟢	🟢
Auvin et al.	2005	🟢	🟢	🟢	🟢	🟢	🟢	🟢
Barton et al.	1989	🟢	🟢	🟢	🟡	🟢	🟢	🟢
Benchimol et al.	2017	🟢	🟢	🟢	🟢	🟢	🟢	🟢
Benchimol et al.	2014	🟢	🟢	🟢	🟢	🟢	🟢	🟢
Bentsen et al.	2002	🟢	🟢	🟢	🟡	🟡	🟢	🟢
Bitton et al.	2014	🟢	🟢	🔴	🔴	🟡	🟢	🟢
Clarkins et al.	1984	🟢	🟡	🟡	🟡	🟡	🟢	🟢
Cosgrove et al.	1996	🟢	🟢	🟡	🔴	🟡	🟢	🟢
El Mouzan	2014	🟢	🟢	🟢	🟢	🟢	🟢	🟢
El-Matary	2014	🟢	🟢	🟢	🟢	🟢	🟢	🟢
Ghione et al.	2018	🟢	🟢	🟡	🟡	🟢	🟢	🟢
Gottrand et al.	1991	🟢	🟢	🟡	🟢	🟢	🟢	🟢
Gower-Rousseau	2009	🟢	🟢	🟡	🟡	🟢	🟢	🟢
Grieci, Büttner	2009	🟢	🟢	🟢	🟢	🟢	🟢	🟢
Henderson et al.	2012	🟢	🟢	🟢	🟢	🟢	🟢	🟢
Hildebrand et al.	1991	🟢	🟢	🟢	🟢	🟢	🟢	🟢
Hildebrand et al.	1994	🟢	🟢	🟢	🟡	🟡	🟢	🟢
Hildebrand et al.	2003	🟢	🟢	🟡	🔴	🟢	🟢	🟢
Hong et al.	2018	🟢	🟢	🟢	🟢	🟢	🟢	🟢
Hope et al.	2012	🟢	🟢	🟢	🟢	🟢	🟡	🟡
Jacobsen, B.A. et al.	2006	🟢	🟢	🟡	🟡	🟡	🟢	🟢
Jacobsen, C. et al.	2008	🟢	🟢	🟢	🟢	🟢	🟢	🟢
Jacobsen, C. et al.	2011	🟡	🟢	🟢	🔴	🔴	🟢	🟢
Karolewska-Bochenek et al.	2009	🟢	🟢	🟢	🟢	🟡	🟢	🟢
Kern et al.	2021	🟢	🟢	🟢	🟢	🟢	🟢	🟢
Kugathasan	2003	🟡	🟢	🟢	🔴	🔴	🟢	🟢
Kuo et al.	2015	🟢	🟢	🟡	🟡	🟢	🟢	🟢
Kwak et al.	2019	🟢	🟢	🟢	🟢	🟡	🟢	🟢
Ladas et al.	2005	🟢	🟢	🟡	🔴	🔴	🟢	🟢
Larsen et al.	2016	🟢	🟢	🟡	🟡	🔴	🟢	🟢
Lethinen et al.	2011	🟢	🟢	🟢	🟢	🟢	🟢	🟢
Lindberg et al.	2000	🟢	🟢	🟡	🟡	🟡	🟢	🟢
Lindquist et al.	1984	🟢	🟢	🟢	🟢	🟢	🟢	🟢
Lopez et al. (2018a)	2018	🟢	🟢	🟢	🟡	🟡	🟢	🟢
Lopez et al.(2018)	2018	🟢	🟢	🟡	🟡	🟡	🟢	🟢
Lovasz et al.	2014	🟢	🟢	🟢	🟢	🟡	🟢	🟢
Malaty et al.	2010	🟡	🟡	🟡	🔴	🟡	🟢	🟢

Malmborg et al.	2013	🟢	🟢	🟢	🟡	🟡	🟢	🟢
Martin-de -Carpi et al.	2013	🟡	🟡	🟡	🔴	🟡	🟢	🟢
Muller et al.	2013	🟢	🟢	🟡	🔴	🟡	🟢	🟢
Olafsdottir et al.	1989	🟢	🟢	🟢	🟢	🟢	🟡	🟢
Ong et al.	2018	🟢	🟢	🟡	🟡	🟡	🟢	🟢
Orel et al.	2009	🟢	🟢	🟡	🟡	🟡	🔴	🟢
Schwarz et al.	2017	🟢	🟢	🟢	🟡	🟡	🟢	🟢
Shivashankar et al.	2017	🟢	🟢	🟢	🟢	🟢	🟢	🟢
Stewenius et al	1995	🟢	🟢	🟡	🔴	🟡	🟡	🟢
Stordal et al.	2004	🟢	🟢	🟢	🟢	🟢	🟢	🟢
Stowe et al.	1990	🟢	🟢	🟢	🟡	🟡	🟢	🟢
Tourtelier et al.	2000	🟢	🟢	🟢	🟡	🟡	🟢	🟢
Turunen et al.	2006	🟢	🟢	🟢	🟡	🟡	🟢	🟢
Urlep et al.	2014	🟢	🟢	🟢	🟡	🟡	🟢	🟢
Urlep et al.	2015	🟢	🟢	🟢	🟡	🟡	🟢	🟢
Urne et al	2002	🟢	🟢	🟢	🟡	🟡	🟡	🟢
van der Zaag_Loonen et al.	2004	🟢	🟢	🟢	🟢	🟡	🟢	🟢
Virta et al.	2017	🟢	🟢	🟢	🟢	🟢	🟢	🟢
Wang et al.	2013	🟢	🟢	🟡	🟡	🟡	🟢	🟢
Watson et al.	2002	🟢	🟢	🟡	🔴	🟡	🟡	🟡
Wittig et al.	2019	🟡	🟡	🟢	🟡	🟢	🟢	🟡
Yamamoto-Furusho et al.	2019	🟢	🟢	🟢	🟡	🟢	🟢	🟢
Yap et al.	2008	🟢	🟢	🟢	🟡	🟡	🟢	🟢
