# Supplementary material for: A systematic review and meta-regression on international trends in the incidence of ulcerative colitis in children and adolescents associated with socioeconomic and geographic factors
Source: Eur J Pediatr. 2024 Jan 17;183(4):1723–32. doi: 10.1007/s00431-024-05428-3 (PMC11001685; doi:10.1007/s00431-024-05428-3)
Supplement: Supplementary file 2 — Supplementary file2 (PDF 57 KB) [file 431_2024_5428_MOESM2_ESM.pdf]

Table summary of included Studies

Nr	Autor	PY	Obs.Per	Start	Continent	Country	Latitude	absLat	Population	Size	IR	SE
1	Abramson et al.	2010	1996-2006	2001	North America	USA	38,581570	4290,07	750000	675	3,426	0,496
2	Adamiak et al	2013	2000-2007	2003	North America	USA	44,430900	4940,49	1300000	675	2,400	
3	Ahmed et al.	2006	1996-2003	1996	Europe	UK	51,481650	5724,50	90000	992	1,500	
4	Armitage et al.	2001	1981-1995	1988	Europe	UK	56,786110	6314,33	1143819	652	1,448	0,233
5	Ashton et al.	2014	2002-2007	2004	Europe	UK	50,902530	5660,10	650000	195397	2,348	0,210
6	Auvin et al.	2005	1988-1999	1993	Europe	FRA	50,528970	5618,57	1312141	7066	2,500	0,000
7	Barton et al.	1989	1968-1983	1975	Europe	UK	55,953350	6221,73	1363573	9688	1,824	0,170
8	Benchimol et al.	2014	1994-2009	2001	North America	Canada	50,000680	5559,82	13500000	2667	2,878	0,826
9	Benchimol et al.	2017	1999-2010	2004	North America	Canada	51,539562	5730,94	26530074	8278	2,690	0,591
10	Bentsen et al.	2002	1990-1993	1991	Europe	Norway	59,103740	6572,04	166456	3062	2,553	0,759
11	Bitton et al.	2014	2001-2008	2004	North America	Canada	52,476090	5835,07	240937	1967	2,260	1,037
12	Clarkins et al.	1984	1960-1979	1970	North America	USA	39,290880	4368,95	2174000	345	1,146	0,273
13	Cosgrove et al.	1996	1983-1993	1988	Europe	UK	51,607680	5738,51	90000	60	0,710	0,000
14	El Mouzan	2014	2003-2012	2007	Asia	SAE	24,631970	2738,95	7128085	323	0,199	0,016
15	El-Matary	2014	1978-2007	1992	North America	Canada	55,001250	6115,86	283167	392	1,423	0,307
16	Ghione et al.	2018	1988-2011	1999	Europe	FRA	50,514410	5616,95	1312141	1225	1,356	0,402
17	Gottrand et al.	1991		1988	Europe	FRA	50,514410	5616,95	1054660	1350	0,500	
18	Gower-Rousseau	2009		1995	Europe	FRA	50,514410	5616,95	1312141	64	0,800	
19	Grieci, Büttner	2009	1997-2006	2001	North America	Canada	50,000680	5559,82	352395	298	8,237	1,327
20	Henderson et al.	2012	1984-1985	1999	Europe	UK	55,953350	6221,73	523984	594	1,603	0,469
21	Hildebrand et al.	1991	1990-2008	1984	Europe	SWE	59,982100	6669,71	130973	542	1,700	0,000
22	Hildebrand et al.	1994	1983-1987	1985	Europe	SWE	57,707230	6416,75	288941	135	2,500	0,561
23	Hildebrand et al.	2003	1990-2001	1995	Europe	SWE	59,325120	6596,65	18000	191	2,170	0,438
24	Hong et al.	2018	2010-2016	2013	Asia	KOR	35,863130	3987,80	974638	124	0,415	0,081
25	Hope et al.	2012		2005	Europe	IRL	53,320230	5928,94	888310	349	1,100	
26	Jacobsen B.A. et al.	2006	1978-2002	1978	Europe	DK	55,939640	6220,20	486274	1217	2,650	0,550
27	Jacobsen, C. et al.	2008	1998-2004	2001	Europe	DK	55,644170	6187,35	430671	503	2,213	0,231
28	Jacobsen, C. et al.	2011		2008	Europe	DK	56,249630	6254,67	668056	50	3,100	
29	Karolewska-Bochenek et al.	2009	2002-2004	2003	Europe	PL	51,988443	5780,85	9000000	491	0,800	0,307
30	Kern et al.	2021	2000-2009	2004	Europe	GER	50,929580	5663,11	436305	344	2,556	0,241

Table summary of included Studies

31	Kugathasan	2003	2000-2001	2000	North America	USA	44,430900	4940,49	540000	491	2,140	
32	Kuo et al.	2015	2000-2010	2005	Asia	TW	23,973940	2665,78	15818502	177	0,215	0,066
33	Kwak et al.	2019	2009-2016	2012	Asia	KOR	36,638390	4074,00	12127750	170	0,236	0,058
34	Ladas	2005		1992	Europe	GRC	39,043900	4341,48	13983	8	7,500	1,992
35	Larsen et al.	2016		2002	Europe	DK	55,670250	6190,25	1000000	1820	5,400	0,878
36	Lehtinen et al.	2011		1995	Europe	FIN	63,246780	7032,72	1100000	1880	6,600	2,500
37	Lindberg et al.	2000	1984-1995	1989	Europe	SWE	59,674970	6635,55	942898	246	3,200	
38	Lindquist et al.	1984	1971-1980	1975	Europe	SWE	59,274730	6591,05	56000	639	0,570	
39	Lopez et al. (2018a)	2018	1996-2015	2005	Australia/Pacific	NZL	-43,494180	-4836,33	110716	156	0,823	0,189
40	Lopez et al.(2018)	2018	2015	2005	Australia/Pacific	NZL	-43,494180	-4836,33	977800	34	1,000	
41	Lovasz et al.	2014	1977-2011	1994	Europe	HUN	47,181760	5246,37	11400	863	2,950	2,250
42	Malaty et al.	2010	1991-2002	1991	North America	USA	29,758940	3309,04	1227491	358	2,160	1,355
43	Malmborg et al.	2013	2002-2007	2005	Europe	SWE	59,325120	6596,65	188437	153	2,771	0,569
44	Martin-de -Carpi et al.	2013	1996-2009	2002	Europe	ESP	39,326230	4372,88	8160000	1712	2,185	0,824
45	Muller et al.	2013	2007-2009	2008	Europe	HUN	47,181760	5246,37	1800000	2107	2,320	
46	Olafsdottir et al.	1989	1984-1985	1984	Europe	Norway	58,936310	6553,42	198570	221	4,300	
47	Ong et al.	2018	1994-2015	2004	Asia	SGP	1,340860	149,10	910761	204	0,367	0,144
48	Orel et al.	2009	1994-2005	2004	Europe	SVN	46,049980	5120,52	283361	167	1,160	0,231
49	Schwarz et al.	2017	2000-2015	2008	Europe	CZ	49,747740	5531,70	82588	261	2,400	0,400
50	Shivashankar et al.	2017	1970-2010	1985	North America	USA	45,989660	5113,82	33501	893	10,553	0,590
51	Stewenius et al	1995		1970	Europe	SWE	55,605290	6183,03	32845	441	6,525	3,063
52	Stordal et al.	2004		1991	Europe	Norway	59,218170	6584,76	174482	471	2,000	
53	Stowe et al.	1990	1940-1989	1970	North America	USA	43,157290	4798,87	460497	1247	12,890	6,597
54	Tourtelier et al.	2000	1994-1997	1995	Europe	FRA	48,162830	5355,46	618049	1377	0,568	0,082
55	Turunen et al.	2006	1987-2003	1995	Europe	FIN	61,498020	6838,27	75706	645	3,006	0,248
56	Urlep et al.	2014	2002-2010	2006	Europe	SVN	46,555630	5176,75	148702	145	2,592	0,316
57	Urlep et al.	2015	2002-2010	2006	Europe	SVN	45,813310	5094,21	414323	260	2,844	0,268
58	Urne et al	2002	1998-2000	1999	Europe	DK	55,867200	6212,15	421898	158	1,767	0,088
59	van der Zaag_Loonen et al.	2004	1999-2001	1999	Europe	NLD	52,500170	5837,75	3873000	98	1,600	
60	Virta et al.	2017	1987-2014	2000	Europe	FIN	63,246780	7032,72	1235000	5379	13,771	1,611
61	Wang et al.	2013	2000-2010	2005	Asia	CHN	31,225300	3472,09	154454	482	2,580	0,485

Table summary of included Studies

62	Watson et al.	2002	1980-1999	1989	Europe	UK	57,148240	6354,59	523984	130	1,100	0,400
63	Wittig et al.	2019	2009-2012	2010	Europe	GER	51,083420	5680,22	1074030	712	6,438	2,227
64	Yamamoto-Furusho et al.	2019	2000-2015	2007	South America	MEX	23,658510	2630,71	40951452	1582	0,020	0,007
65	Yap et al.	2008	2002-2003	2003	Australia/Pacific	NZL	-41,500080	-4614,60	885000	2073	0,500	
