# Supplementary material for: A systematic review and meta-regression on international trends in the incidence of ulcerative colitis in children and adolescents associated with socioeconomic and geographic factors
Source: Eur J Pediatr. 2024 Jan 17;183(4):1723–32. doi: 10.1007/s00431-024-05428-3 (PMC11001685; doi:10.1007/s00431-024-05428-3)
Supplement: Supplementary file 3 — Supplementary file3 (DOCX 2260 KB) [file 431_2024_5428_MOESM3_ESM.docx]

**Supplement Systematic literature search strategy**

PubMed search strategy

1. colitis, ulcerative[MeSH Terms]
2. OR crohn disease[MeSH Terms]
3. OR Crohn Disease[Text Word
4. OR Crohn´s Disease[Text Word]
5. OR inflammatory bowel diseases[MeSH Terms]
6. OR Inflammatory Bowel Disease*[Text Word]
7. AND
8. (incidence[MeSH Terms]
9. OR incidence[Text Word]
10. OR prevalence[MeSH Terms]
11. OR prevalence[Text Word]
12. AND
13. 1970:2019[pdat]
14. AND
15. "humans"[MeSH Terms]
16. AND
17. "infant"[MeSH Terms]
18. OR "child"[MeSH Terms]
19. OR "adolescent"[MeSH Terms]
20. OR "infant"[MeSH Terms]
21. OR "infant"[MeSH Terms:noexp]
22. OR "child, preschool"[MeSH Terms]
23. OR "child"[MeSH Terms:noexp]
24. OR "adolescent"[MeSH Terms]

## EMBASE via Ovid search strategy

1. colitis, [ulcerative.mp](http://ulcerative.mp). or exp ulcerative colitis/
2. crohn [disease.mp](http://disease.mp). or exp Crohn disease/
3. Inflammatory Bowel [Disease.mp](http://Disease.mp). or exp inflammatory bowel disease/
4. exp standardized incidence ratio/ and [incidence.mp](http://incidence.mp). and exp incidence/
5. [prevalence.mp](http://prevalence.mp). or exp prevalence/
6. 1 or 2 or 3
7. 4 and 6
8. 4 or 5
9. limit 8 to (human and yr="1970 - 2019" and (infant <to one year> or child <unspecified age> or preschool child <1 to 6 years> or school child <7 to 12 years> or adolescent <13 to 17 years>))

**Supplement Results systematic literature search**


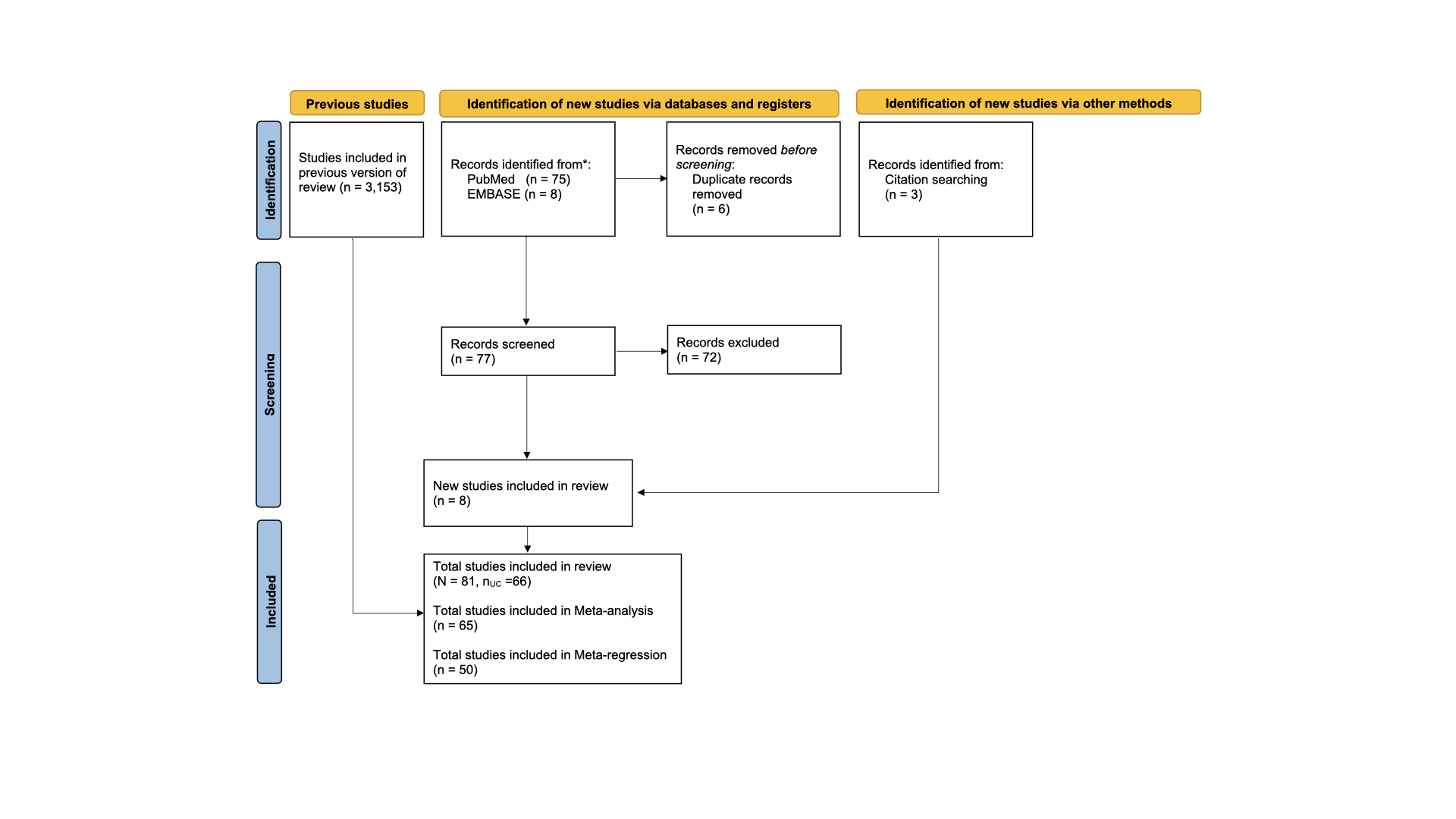


**Fig.S 1:** PRISMA 2020 flow diagram for updated systematic reviews which included searches of databases, registers and other sources, (21)

**Supplement References of the found studies**

Abramson, O., M. Durant, W. Mow, A. Finley, P. Kodali, A. Wong, V. Tavares, u. a. „Incidence, Prevalence, and Time Trends of Pediatric Inflammatory Bowel Disease in Northern California, 1996 to 2006“. J Pediatr 157, Nr. 2 (August 2010): 233-239.e1. <https://doi.org/10.1016/j.jpeds.2010.02.024>.

Adamiak, T., D. Walkiewicz-Jedrzejczak, D. Fish, C. Brown, J. Tung, K. Khan, W. Faubion Jr., u. a. „Incidence, Clinical Characteristics, and Natural History of Pediatric IBD in Wisconsin: A Population-Based Epidemiological Study“. Inflamm Bowel Dis 19, Nr. 6 (Mai 2013): 1218–23. <https://doi.org/10.1097/MIB.0b013e318280b13e>.

Ahmed, M., I. H. Davies, K. Hood, und H. R. Jenkins. „Incidence of Paediatric Inflammatory Bowel Disease in South Wales“. Arch Dis Child 91, Nr. 4 (April 2006): 344–45. <https://doi.org/10.1136/adc.2004.067413>.

Armitage, E., H. E. Drummond, D. C. Wilson, und S. Ghosh. „Increasing Incidence of Both Juvenile-Onset Crohn’s Disease and Ulcerative Colitis in Scotland“. Eur J Gastroenterol Hepatol 13, Nr. 12 (Dezember 2001): 1439–47.

Ashton, J. J., A. E. Wiskin, S. Ennis, A. Batra, N. A. Afzal, und R. M. Beattie. „Rising Incidence of Paediatric Inflammatory Bowel Disease (PIBD) in Wessex, Southern England“. Arch Dis Child 99, Nr. 7 (Juli 2014): 659–64. <https://doi.org/10.1136/archdischild-2013-305419>.

Auvin, Stéphane, Florence Molinié, Corinne Gower-Rousseau, Franck Brazier, Véronique Merle, Bruno Grandbastien, Raymond Marti, u. a. „Incidence, Clinical Presentation and Location at Diagnosis of Pediatric Inflammatory Bowel Disease: A Prospective Population-Based Study in Northern {France} (1988-1999).“ Journal of Pediatric Gastroenterology and Nutrition 41, Nr. 1 (Juli 2005): 49–55. <https://doi.org/10.1097/01.mpg.0000162479.74277.86>.

Barton, J. R., S. Gillon, und A. Ferguson. „Incidence of Inflammatory Bowel Disease in Scottish Children between 1968 and 1983; Marginal Fall in Ulcerative Colitis, Three-Fold Rise in Crohn’s Disease“. Gut 30, Nr. 5 (Mai 1989): 618–22. <https://doi.org/10.1136/gut.30.5.618>.

Benchimol, E. I., C. N. Bernstein, A. Bitton, M. W. Carroll, H. Singh, A. R. Otley, M. Vutcovici, u. a. „Trends in Epidemiology of Pediatric Inflammatory Bowel Disease in Canada: Distributed Network Analysis of Multiple Population-Based Provincial Health Administrative Databases“. Am J Gastroenterol 112, Nr. 7 (Juli 2017): 1120–34. <https://doi.org/10.1038/ajg.2017.97>.

Benchimol, E. I., D. R. Mack, G. C. Nguyen, S. B. Snapper, W. Li, N. Mojaverian, P. Quach, und A. M. Muise. „Incidence, Outcomes, and Health Services Burden of Very Early Onset Inflammatory Bowel Disease“. Gastroenterology 147, Nr. 4 (Oktober 2014): 803-813.e7; quiz e14-5. <https://doi.org/10.1053/j.gastro.2014.06.023>.

Bentsen, B. S., B. Moum, und A. Ekbom. „Incidence of Inflammatory Bowel Disease in Children in Southeastern Norway: A Prospective Population-Based Study 1990-94“. Scand J Gastroenterol 37, Nr. 5 (Mai 2002): 540–45. <https://doi.org/10.1080/00365520252903080>.

Bitton, A., M. Vutcovici, V. Patenaude, M. Sewitch, S. Suissa, und P. Brassard. „Epidemiology of Inflammatory Bowel Disease in Quebec: Recent Trends“. Inflamm Bowel Dis 20, Nr. 10 (Oktober 2014): 1770–76. <https://doi.org/10.1097/mib.0000000000000162>.

Calkins, B. M., A. M. Lilienfeld, C. F. Garland, und A. I. Mendeloff. „Trends in Incidence Rates of Ulcerative Colitis and Crohn’s Disease“. Dig Dis Sci 29, Nr. 10 (Oktober 1984): 913–20. <https://doi.org/10.1007/bf01312480>.

Cosgrove, M., R. F. Al-Atia, und H. R. Jenkins. „The Epidemiology of Paediatric Inflammatory Bowel Disease“. Arch Dis Child 74, Nr. 5 (Mai 1996): 460–61. <https://doi.org/10.1136/adc.74.5.460>.

El Mouzan, M. I., O. Saadah, K. Al-Saleem, M. Al Edreesi, M. Hasosah, A. Alanazi, M. Al Mofarreh, u. a. „Incidence of Pediatric Inflammatory Bowel Disease in Saudi Arabia: A Multicenter National Study“. Inflamm Bowel Dis 20, Nr. 6 (Juni 2014): 1085–90. https://doi.org/10.1097/mib.0000000000000048.

El-Matary, W., S. P. Moroz, und C. N. Bernstein. „Inflammatory Bowel Disease in Children of Manitoba: 30 Years’ Experience of a Tertiary Center“. J Pediatr Gastroenterol Nutr 59, Nr. 6 (Dezember 2014): 763–66. <https://doi.org/10.1097/mpg.0000000000000525>.

Ghione, S., H. Sarter, M. Fumery, L. Armengol-Debeir, G. Savoye, D. Ley, C. Spyckerelle, u. a. „Dramatic Increase in Incidence of Ulcerative Colitis and Crohn’s Disease (1988-2011): A Population-Based Study of French Adolescents“. Am J Gastroenterol 113, Nr. 2 (Februar 2018): 265–72. <https://doi.org/10.1038/ajg.2017.228>.

Gottrand, F., J. F. Colombel, L. Moreno, J. L. Salomez, J. P. Farriaux, und A. Cortot. „[Incidence of inflammatory bowel diseases in children in the Nord-Pas-de-Calais region]“. Arch Fr Pediatr 48, Nr. 1 (Januar 1991): 25–28.

Gower-Rousseau, C., L. Dauchet, G. Vernier-Massouille, E. Tilloy, F. Brazier, V. Merle, J. L. Dupas, u. a. „The Natural History of Pediatric Ulcerative Colitis: A Population-Based Cohort Study“. Am J Gastroenterol 104, Nr. 8 (August 2009): 2080–88. <https://doi.org/10.1038/ajg.2009.177>.

Grieci, T., und A. Butter. „The Incidence of Inflammatory Bowel Disease in the Pediatric Population of Southwestern Ontario“. J Pediatr Surg 44, Nr. 5 (Mai 2009): 977–80. <https://doi.org/10.1016/j.jpedsurg.2009.01.038>.

Henderson, P., R. Hansen, F. L. Cameron, K. Gerasimidis, P. Rogers, W. M. Bisset, E. L. Reynish, u. a. „Rising Incidence of Pediatric Inflammatory Bowel Disease in Scotland“. Inflamm Bowel Dis 18, Nr. 6 (Juni 2012): 999–1005. <https://doi.org/10.1002/ibd.21797>.

Hildebrand, H., M. Brydolf, L. Holmquist, I. Krantz, und B. Kristiansson. „Incidence and Prevalence of Inflammatory Bowel Disease in Children in South-Western Sweden“. Acta Paediatr 83, Nr. 6 (Juni 1994): 640–45. <https://onlinelibrary.wiley.com/doi/abs/10.1111/j.1651-2227.1994.tb13098.x?sid=nlm%3Apubmed>.

Hildebrand, H., Y. Finkel, L. Grahnquist, J. Lindholm, A. Ekbom, und J. Askling. „Changing Pattern of Paediatric Inflammatory Bowel Disease in Northern Stockholm 1990-2001“. Gut 52, Nr. 10 (Oktober 2003): 1432–34. <https://doi.org/10.1136/gut.52.10.1432>.

Hildebrand, H., B. Fredrikzon, L. Holmquist, B. Kristiansson, und B. Lindquist. „Chronic Inflammatory Bowel Disease in Children and Adolescents in Sweden“. J Pediatr Gastroenterol Nutr 13, Nr. 3 (Oktober 1991): 293–97. <https://doi.org/10.1097/00005176-199110000-00010>.

Hong, S. J., S. M. Cho, B. H. Choe, H. J. Jang, K. H. Choi, B. Kang, J. E. Kim, und J. H. Hwang. „Characteristics and Incidence Trends for Pediatric Inflammatory Bowel Disease in Daegu-Kyungpook Province in Korea: A Multi-Center Study“. J Korean Med Sci 33, Nr. 18 (30. April 2018): e132. <https://doi.org/10.3346/jkms.2018.33.e132>.

Hope, B., R. Shahdadpuri, C. Dunne, A. M. Broderick, T. Grant, M. Hamzawi, K. O’Driscoll, S. Quinn, S. Hussey, und B. Bourke. „Rapid Rise in Incidence of Irish Paediatric Inflammatory Bowel Disease“. Arch Dis Child 97, Nr. 7 (Juli 2012): 590–94. <https://doi.org/10.1136/archdischild-2011-300651>.

Jacobsen, B. A., J. Fallingborg, H. H. Rasmussen, K. R. Nielsen, A. M. Drewes, E. Puho, G. L. Nielsen, und H. T. Sorensen. „Increase in Incidence and Prevalence of Inflammatory Bowel Disease in Northern Denmark: A Population-Based Study, 1978-2002“. Eur J Gastroenterol Hepatol 18, Nr. 6 (Juni 2006): 601–6. <https://doi.org/10.1097/00042737-200606000-00005>.

Jakobsen, C., A. Paerregaard, P. Munkholm, J. Faerk, A. Lange, J. Andersen, M. Jakobsen, I. Kramer, J. Czernia-Mazurkiewicz, und V. Wewer. „Pediatric Inflammatory Bowel Disease: Increasing Incidence, Decreasing Surgery Rate, and Compromised Nutritional Status: A Prospective Population-Based Cohort Study 2007-2009“. Inflamm Bowel Dis 17, Nr. 12 (Dezember 2011): 2541–50. <https://doi.org/10.1002/ibd.21654>.

Jakobsen, C., V. Wewer, F. Urne, J. Andersen, J. Faerk, I. Kramer, B. Stagegaard, B. Pilgaard, B. Weile, und A. Paerregaard. „Incidence of ulcerative colitis and Crohn’s disease in Danish children: Still rising or levelling out?“ Journal of Crohn’s and Colitis 2, Nr. 2 (2008): 152–57. https://doi.org/10.1016/j.crohns.2008.01.006.

Karolewska-Bochenek, K., I. Lazowska-Przeorek, P. Albrecht, K. Grzybowska, J. Ryzko, K. Szamotulska, A. Radzikowski, u. a. „Epidemiology of inflammatory bowel disease among children in Poland“. Digestion 79, Nr. 2 (2009): 121–29. <https://doi.org/10.1159/000209382>.

Kern, Ivana, Olaf Schoffer, Wieland Kiess, Jobst Henker, Martin W. Laaß, Ulf Winkler, Jürgen Quietzsch, u. a. „Incidence Trends of Pediatric Onset Inflammatory Bowel Disease in the Years 2000-2009 in Saxony, Germany-First Results of the Saxon Pediatric IBD Registry“. PloS One 16, Nr. 1 (2021): e0243774. <https://doi.org/10.1371/journal.pone.0243774>.

Kugathasan, S., R. H. Judd, R. G. Hoffmann, J. Heikenen, G. Telega, F. Khan, S. Weisdorf-Schindele, u. a. „Epidemiologic and Clinical Characteristics of Children with Newly Diagnosed Inflammatory Bowel Disease in Wisconsin: A Statewide Population-Based Study“. J Pediatr 143, Nr. 4 (Oktober 2003): 525–31. <https://doi.org/10.1067/s0022-3476(03)00444-x>.

Kuo, C. J., K. H. Yu, L. C. See, C. T. Chiu, M. Y. Su, C. M. Hsu, C. F. Kuo, M. J. Chiou, J. R. Liu, und H. W. Wang. „The Trend of Inflammatory Bowel Diseases in Taiwan: A Population-Based Study“. Dig Dis Sci 60, Nr. 8 (August 2015): 2454–62. <https://doi.org/10.1007/s10620-015-3630-z>.

Kwak, M. S., J. M. Cha, H. H. Lee, Y. S. Choi, S. I. Seo, K. J. Ko, D. I. Park, S. H. Kim, und T. J. Kim. „Emerging trends of inflammatory bowel disease in South Korea: A nationwide population-based study“. Journal of Gastroenterology and Hepatology (Australia) 34, Nr. 6 (2019): 1018–26. https://doi.org/10.1111/jgh.14542.

Ladas, S. D., E. Mallas, K. Giorgiotis, G. Karamanolis, D. Trigonis, A. Markadas, V. Sipsa, und S. A. Raptis. „Incidence of Ulcerative Colitis in Central Greece: A Prospective Study“. World J Gastroenterol 11, Nr. 12 (28. März 2005): 1785–87. <https://doi.org/10.3748/wjg.v11.i12.1785>.

Larsen, M. D., M. E. Baldal, R. G. Nielsen, J. Nielsen, K. Lund, und B. M. Norgard. „The Incidence of Crohn’s Disease and Ulcerative Colitis since 1995 in Danish Children and Adolescents <17 Years - Based on Nationwide Registry Data“. Scand J Gastroenterol 51, Nr. 9 (September 2016): 1100–1105. <https://doi.org/10.3109/00365521.2016.1172340>.

Lehtinen, Pieta, Merja Ashorn, Sari Iltanen, Raimo Jauhola, Pekka Jauhonen, Kaija-Leena Kolho, und Anssi Auvinen. „Incidence Trends of Pediatric Inflammatory Bowel Disease in Finland, 1987-2003, a Nationwide Study.“ Inflammatory Bowel Diseases 17, Nr. 8 (August 2011): 1778–83. <https://doi.org/10.1002/ibd.21550>.

Lindberg, E., B. Lindquist, L. Holmquist, und H. Hildebrand. „Inflammatory Bowel Disease in Children and Adolescents in Sweden, 1984-1995“. J Pediatr Gastroenterol Nutr 30, Nr. 3 (März 2000): 259–64. <https://doi.org/10.1097/00005176-200003000-00009>.

Lindquist, B. L., G. Jarnerot, und G. Wickbom. „Clinical and Epidemiological Aspects of Crohn’s Disease in Children and Adolescents“. Scand J Gastroenterol 19, Nr. 4 (Juni 1984): 502–6.

Lopez, R. N., L. Appleton, R. B. Gearry, und A. S. Day. „Rising Incidence of Paediatric Inflammatory Bowel Disease in Canterbury, New Zealand, 1996-2015“. Journal of Pediatric Gastroenterology and Nutrition 66, Nr. 2 (2018): e45–50. <https://doi.org/10.1097/MPG.0000000000001688>.

Lopez, R. N., H. M. Evans, L. Appleton, J. Bishop, S. Chin, S. Mouat, R. B. Gearry, und A. S. Day. „Prospective Incidence of Paediatric Inflammatory Bowel Disease in New Zealand in 2015: Results from the Paediatric Inflammatory Bowel Disease in New Zealand (PINZ) Study“. Journal of Pediatric Gastroenterology and Nutrition 66, Nr. 5 (2018): e122–26. <https://doi.org/10.1097/MPG.0000000000001806>.

Lovasz, B. D., L. Lakatos, A. Horvath, T. Pandur, Z. Erdelyi, M. Balogh, I. Szipocs, u. a. „Incidence Rates and Disease Course of Paediatric Inflammatory Bowel Diseases in Western Hungary between 1977 and 2011“. Dig Liver Dis 46, Nr. 5 (Mai 2014): 405–11. <https://doi.org/10.1016/j.dld.2013.12.013>.

Malaty, H. M., X. Fan, A. R. Opekun, C. Thibodeaux, und G. D. Ferry. „Rising Incidence of Inflammatory Bowel Disease among Children: A 12-Year Study“. J Pediatr Gastroenterol Nutr 50, Nr. 1 (Januar 2010): 27–31. https://doi.org/10.1097/MPG.0b013e3181b99baa.

Malmborg, P., L. Grahnquist, J. Lindholm, S. Montgomery, und H. Hildebrand. „Increasing Incidence of Paediatric Inflammatory Bowel Disease in Northern Stockholm County, 2002-2007“. J Pediatr Gastroenterol Nutr 57, Nr. 1 (Juli 2013): 29–34. <https://doi.org/10.1097/MPG.0b013e31828f21b4>.

Martin-de-Carpi, J., E. Ramos, S. Jimenez, M. J. Martinez-Gomez, E. Medina, J. Serrano, E. Ricart, u. a. „Increasing incidence of pediatric inflammatory bowel disease in Spain (1996-2009): The SPIRIT registry“. Inflammatory Bowel Diseases 19, Nr. 1 (2013): 73–80. <https://doi.org/10.1002/ibd.22980>.

Muller, K. E., P. L. Lakatos, A. Arato, J. B. Kovacs, A. Varkonyi, D. Szucs, E. Szakos, u. a. „Incidence, Paris Classification, and Follow-up in a Nationwide Incident Cohort of Pediatric Patients with Inflammatory Bowel Disease“. J Pediatr Gastroenterol Nutr 57, Nr. 5 (November 2013): 576–82. <https://doi.org/10.1097/MPG.0b013e31829f7d8c>.

Olafsdottir, E. J., G. Fluge, und K. Haug. „Chronic Inflammatory Bowel Disease in Children in Western Norway“. J Pediatr Gastroenterol Nutr 8, Nr. 4 (Mai 1989): 454–58. <https://doi.org/10.1097/00005176-198905000-00006>.

Ong, C., M. M. Aw, M. J. Liwanag, S. H. Quak, und K. B. Phua. „Rapid Rise in the Incidence and Clinical Characteristics of Pediatric Inflammatory Bowel Disease in a South-East Asian Cohort in Singapore, 1994-2015“. J Dig Dis 19, Nr. 7 (Juli 2018): 395–403. <https://doi.org/10.1111/1751-2980.12641>.

Orel, R., T. Kamhi, G. Vidmar, und P. Mamula. „Epidemiology of Pediatric Chronic Inflammatory Bowel Disease in Central and Western Slovenia, 1994-2005“. J Pediatr Gastroenterol Nutr 48, Nr. 5 (Mai 2009): 579–86. <https://doi.org/10.1097/MPG.0b013e318164d903>.

Schwarz, J., J. Sykora, D. Cvalinova, R. Pomahacova, J. Kleckova, M. Kryl, und P. Vcelak. „Inflammatory Bowel Disease Incidence in Czech Children: A Regional Prospective Study, 2000-2015“. World J Gastroenterol 23, Nr. 22 (14. Juni 2017): 4090–4101. <https://doi.org/10.3748/wjg.v23.i22.4090>.

Shivashankar, Raina, William J. Tremaine, W. Scott Harmsen, und Edward V. Jr Loftus. „Incidence and Prevalence of Crohn’s Disease and Ulcerative Colitis in Olmsted County, Minnesota From 1970 Through 2010.“ Clinical Gastroenterology and Hepatology : The Official Clinical Practice Journal of the American Gastroenterological Association 15, Nr. 6 (Juni 2017): 857–63. <https://doi.org/10.1016/j.cgh.2016.10.039>.

Stewenius, J., I. Adnerhill, G. Ekelund, C. H. Floren, F. T. Fork, L. Janzon, C. Lindstrom, I. Mars, M. Nyman, und J. E. Rosengren. „Ulcerative Colitis and Indeterminate Colitis in the City of Malmo, Sweden. A 25-Year Incidence Study“. Scand J Gastroenterol 30, Nr. 1 (Januar 1995): 38–43. <https://doi.org/10.3109/00365529509093233>.

Stordal, K., J. Jahnsen, B. S. Bentsen, und B. Moum. „Pediatric Inflammatory Bowel Disease in Southeastern Norway: A Five-Year Follow-up Study“. Digestion 70, Nr. 4 (2004): 226–30. https://doi.org/10.1159/000082893.

Stowe, S. P., S. R. Redmond, J. M. Stormont, A. N. Shah, L. N. Chessin, H. L. Segal, und W. Y. Chey. „An Epidemiologic Study of Inflammatory Bowel Disease in Rochester, New York. Hospital Incidence“. Gastroenterology 98, Nr. 1 (Januar 1990): 104–10. <https://doi.org/10.1016/0016-5085(90)91297-j>.

Tourtelier, Y., A. Dabadie, I. Tron, J. L. Alexandre, M. Robaskiewicz, E. Cruchant, J. A. Seyrig, D. Heresbach, und J. F. Bretagne. „[Incidence of inflammatory bowel disease in children in Brittany (1994-1997). Breton association of study and research on digestive system diseases (Abermad)]“. Arch Pediatr 7, Nr. 4 (April 2000): 377–84. <https://doi.org/10.1016/s0929-693x(00)88832-6>.

Turunen, P., K. L. Kolho, A. Auvinen, S. Iltanen, H. Huhtala, und M. Ashorn. „Incidence of Inflammatory Bowel Disease in Finnish Children, 1987-2003“. Inflamm Bowel Dis 12, Nr. 8 (August 2006): 677–83. <https://doi.org/10.1097/00054725-200608000-00002>.

Urlep, D., R. Blagus, und R. Orel. „Incidence Trends and Geographical Variability of Pediatric Inflammatory Bowel Disease in Slovenia: A Nationwide Study“. Biomed Res Int 2015 (2015): 921730. <https://doi.org/10.1155/2015/921730>.

Urlep, D., T. K. Trop, R. Blagus, und R. Orel. „Incidence and Phenotypic Characteristics of Pediatric IBD in Northeastern Slovenia, 2002-2010“. J Pediatr Gastroenterol Nutr 58, Nr. 3 (März 2014): 325–32. <https://doi.org/10.1097/mpg.0000000000000207>.

Urne, F. U., und A. Paerregaard. „[Chronic inflammatory bowel disease in children. An epidemiological study from eastern Denmark 1998-2000]“. Ugeskr Laeger 164, Nr. 49 (2. Dezember 2002): 5810–14.

Vicentin, R., M. Wagener, A. B. Pais, M. Contreras, und M. Orsi. „One-Year Prospective Registry of Inflammatory Bowel Disease in the Argentine Pediatric Population“. Arch Argent Pediatr 115, Nr. 6 (1. Dezember 2017): 533–40. <https://doi.org/10.5546/aap.2017.eng.533>.

Virta, L. J., M. M. Saarinen, und K. L. Kolho. „Inflammatory Bowel Disease Incidence Is on the Continuous Rise Among All Paediatric Patients Except for the Very Young: A Nationwide Registry-Based Study on 28-Year Follow-Up“. J Crohns Colitis 11, Nr. 2 (Februar 2017): 150–56. <https://doi.org/10.1093/ecco-jcc/jjw148>.

Wang, X. Q., Y. Zhang, C. D. Xu, L. R. Jiang, Y. Huang, H. M. Du, und X. J. Wang. „Inflammatory Bowel Disease in Chinese Children: A Multicenter Analysis over a Decade from Shanghai“. Inflamm Bowel Dis 19, Nr. 2 (Februar 2013): 423–28. <https://doi.org/10.1097/MIB.0b013e318286f9f2>.

Watson, A. J., A. T. Johnston, P. M. Barker, G. G. Youngson, W. M. Bisset, und A. A. Mahomed. „The Presentation and Management of Juvenile-Onset Chronic Inflammatory Bowel Disease in Northeastern Scotland“. J Pediatr Surg 37, Nr. 1 (Januar 2002): 83–86. <https://doi.org/10.1053/jpsu.2002.29434>.

Wittig, Regina, Lucia Albers, Sibylle Koletzko, Joachim Saam, und Rüdiger von Kries. „Pediatric Chronic Inflammatory Bowel Disease in a German Statutory Health INSURANCE-Incidence Rates From 2009 to 2012.“ Journal of Pediatric Gastroenterology and Nutrition 68, Nr. 2 (Februar 2019): 244–50. <https://doi.org/10.1097/MPG.0000000000002162>.

Yamamoto-Furusho, J. K., A. Sarmiento-Aguilar, J. J. Toledo-Maurino, K. E. Bozada-Gutierrez, F. J. Bosques-Padilla, M. A. Martinez-Vazquez, V. Marroquin-Jimenez, u. a. „Incidence and Prevalence of Inflammatory Bowel Disease in Mexico from a Nationwide Cohort Study in a Period of 15 Years (2000-2017)“. Medicine (Baltimore) 98, Nr. 27 (Juli 2019): e16291. <https://doi.org/10.1097/md.0000000000016291>.

Yap, J., A. Wesley, S. Mouat, und S. Chin. „Paediatric Inflammatory Bowel Disease in New Zealand“. N Z Med J 121, Nr. 1283 (3. Oktober 2008): 19–34.

Zaag-Loonen, H. J. van der, M. Casparie, J. A. Taminiau, J. C. Escher, R. R. Pereira, und H. H. Derkx. „The Incidence of Pediatric Inflammatory Bowel Disease in the Netherlands: 1999-2001“. J Pediatr Gastroenterol Nutr 38, Nr. 3 (März 2004): 302–7. https://doi.org/10.1097/00005176-200403000-00014.

# Supplement Summary of the included studies (see PDF)

# Supplement Risk of Bias Analysis (see PDF)

# Supplement Meta-analysis of all studies (raw data)


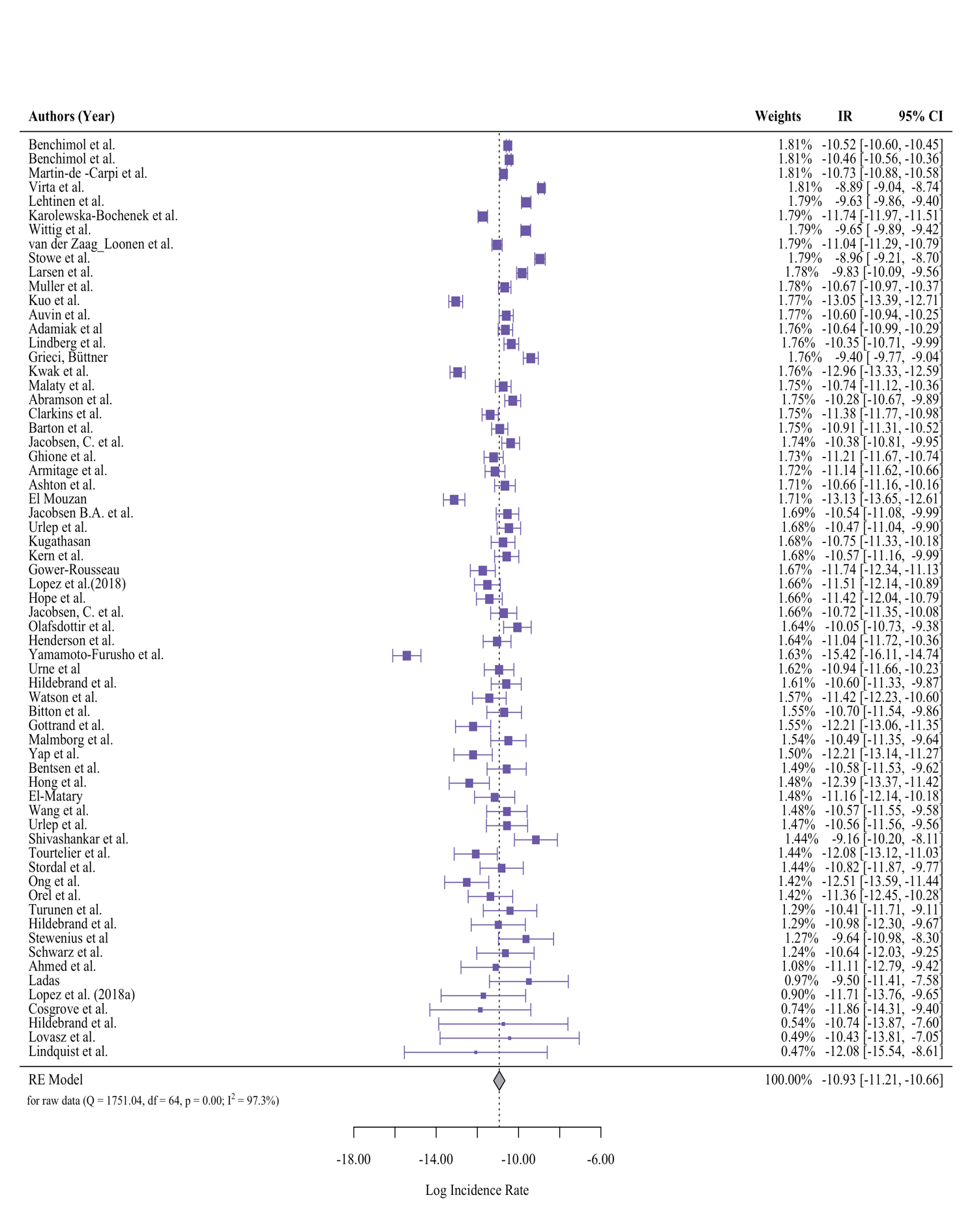


**Fig.S 2:** Forrest-plot all Studies UC

# Supplement Subgroup analysis in 10-year steps


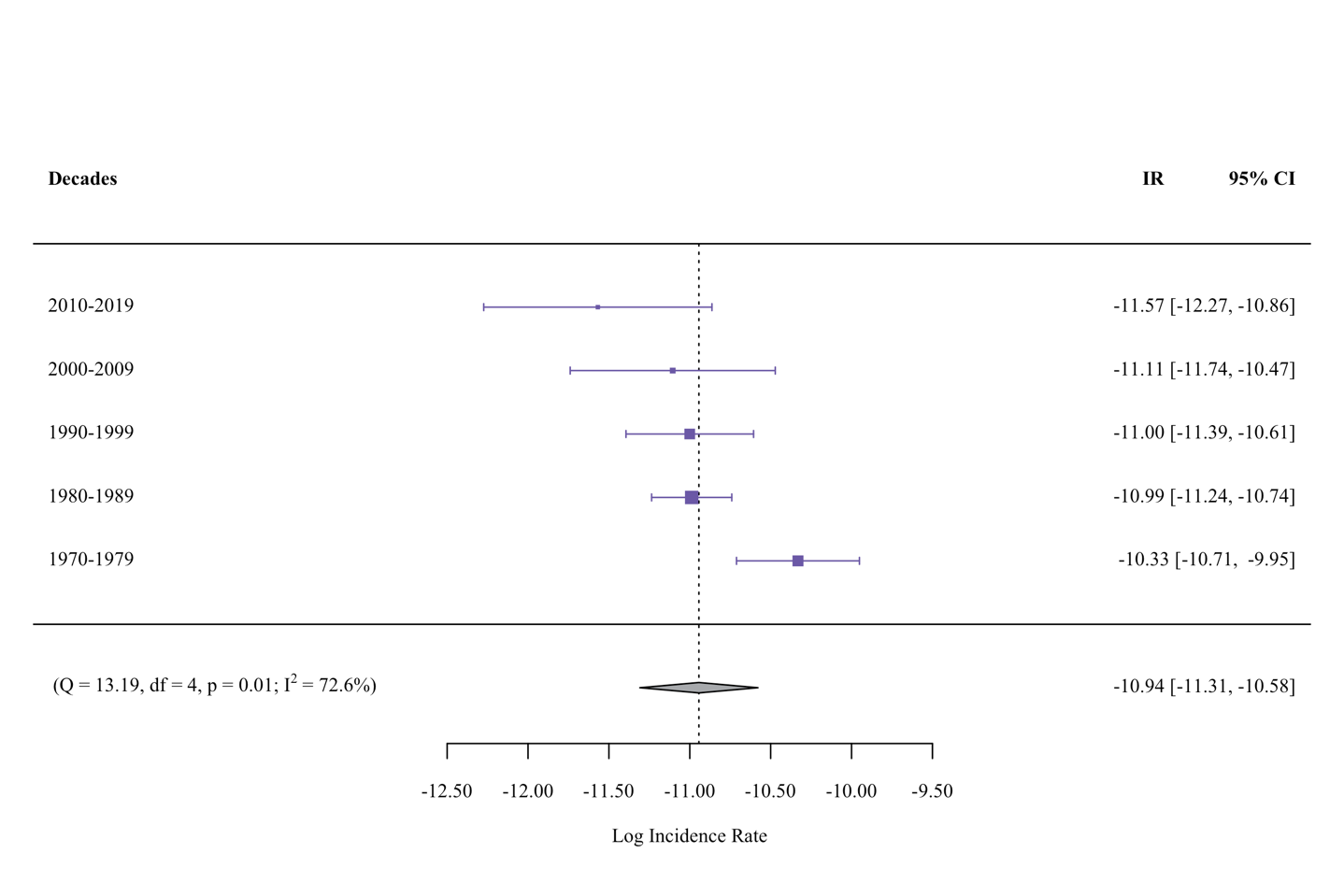


**Fig.S 3:** Forest Plot pooled incidence rates per 10 years


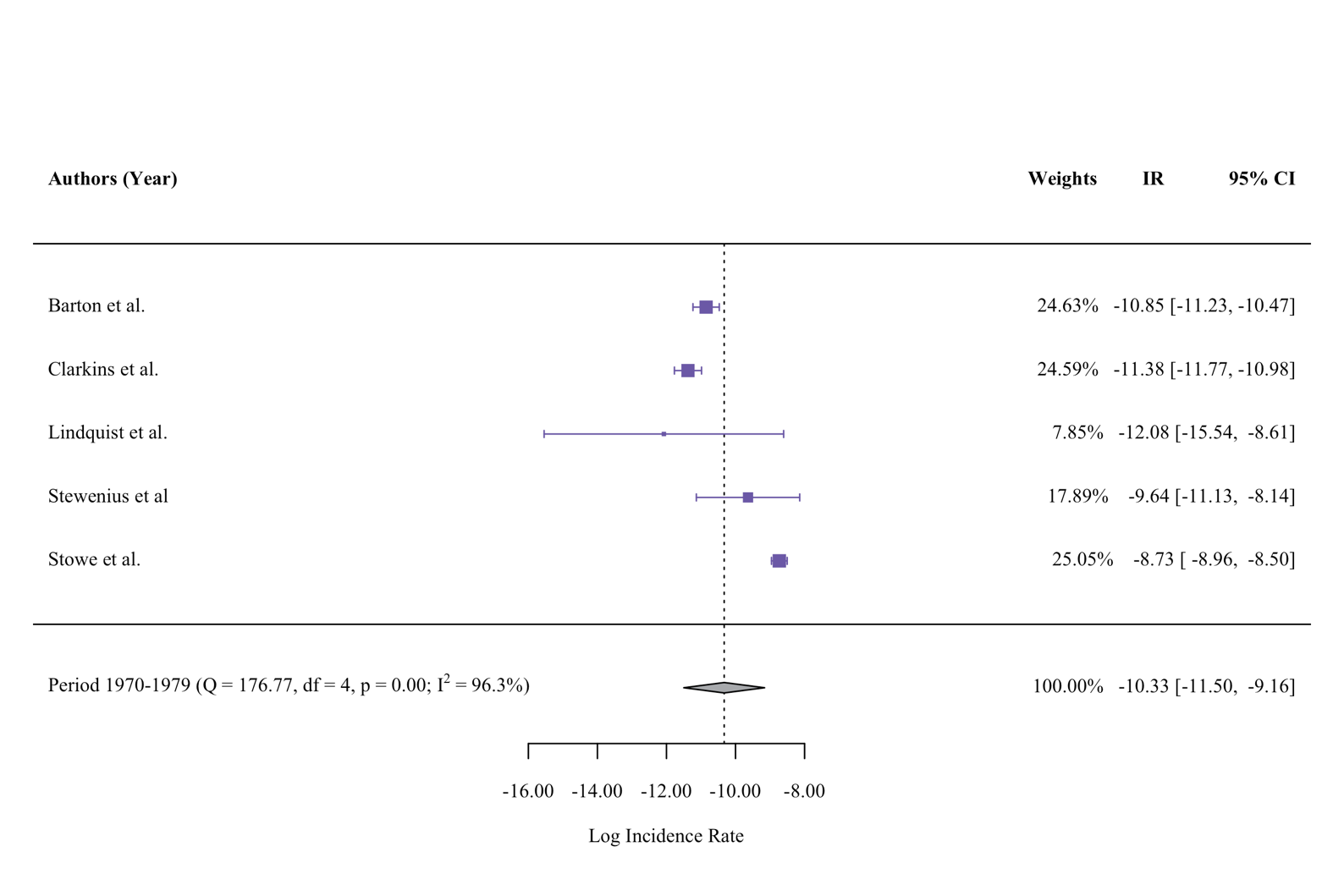


**Fig.S 4:** Forest Plot Studies period 1970-1979


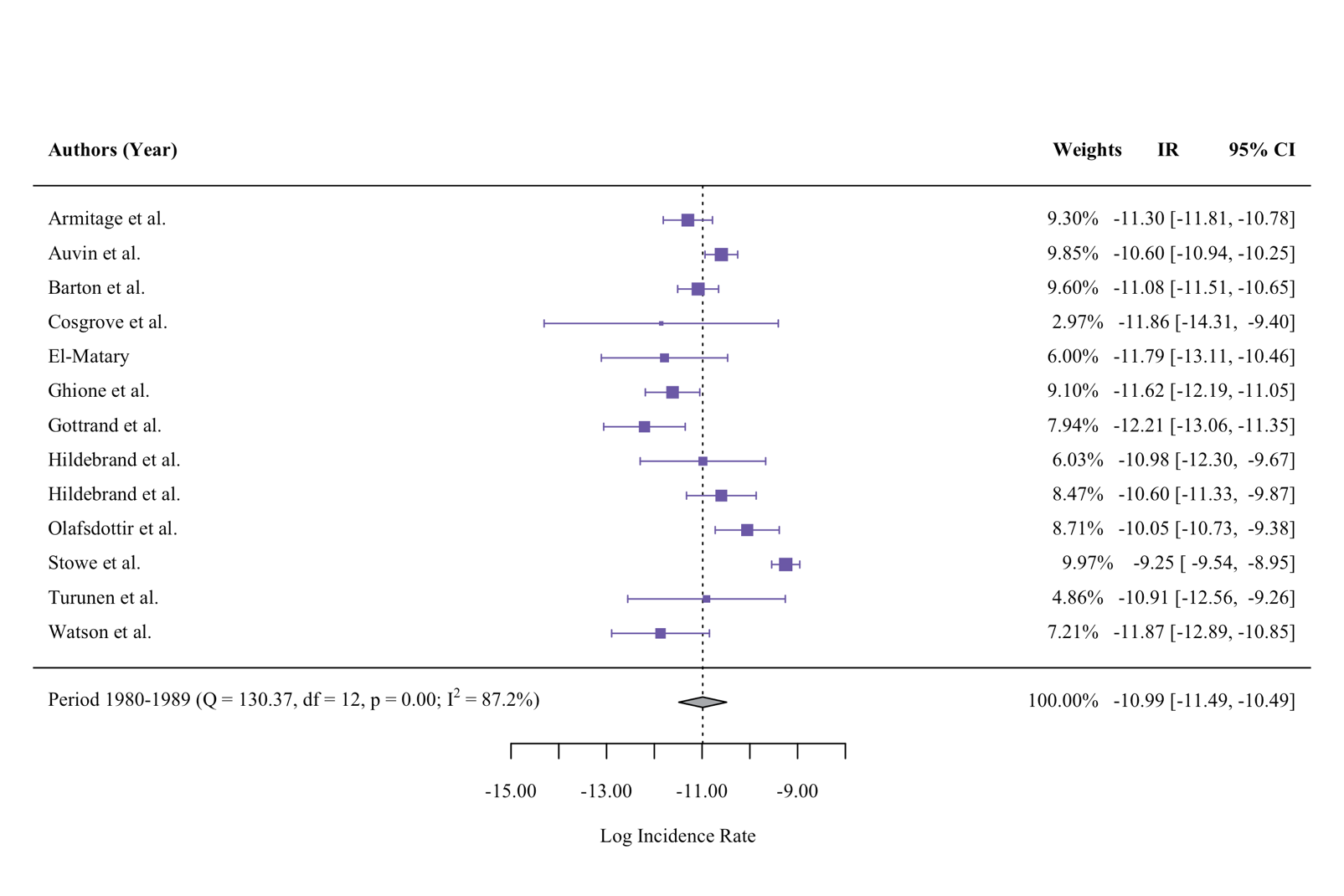


**Fig.S 5:** Forest Plot Studies period 1980-1989


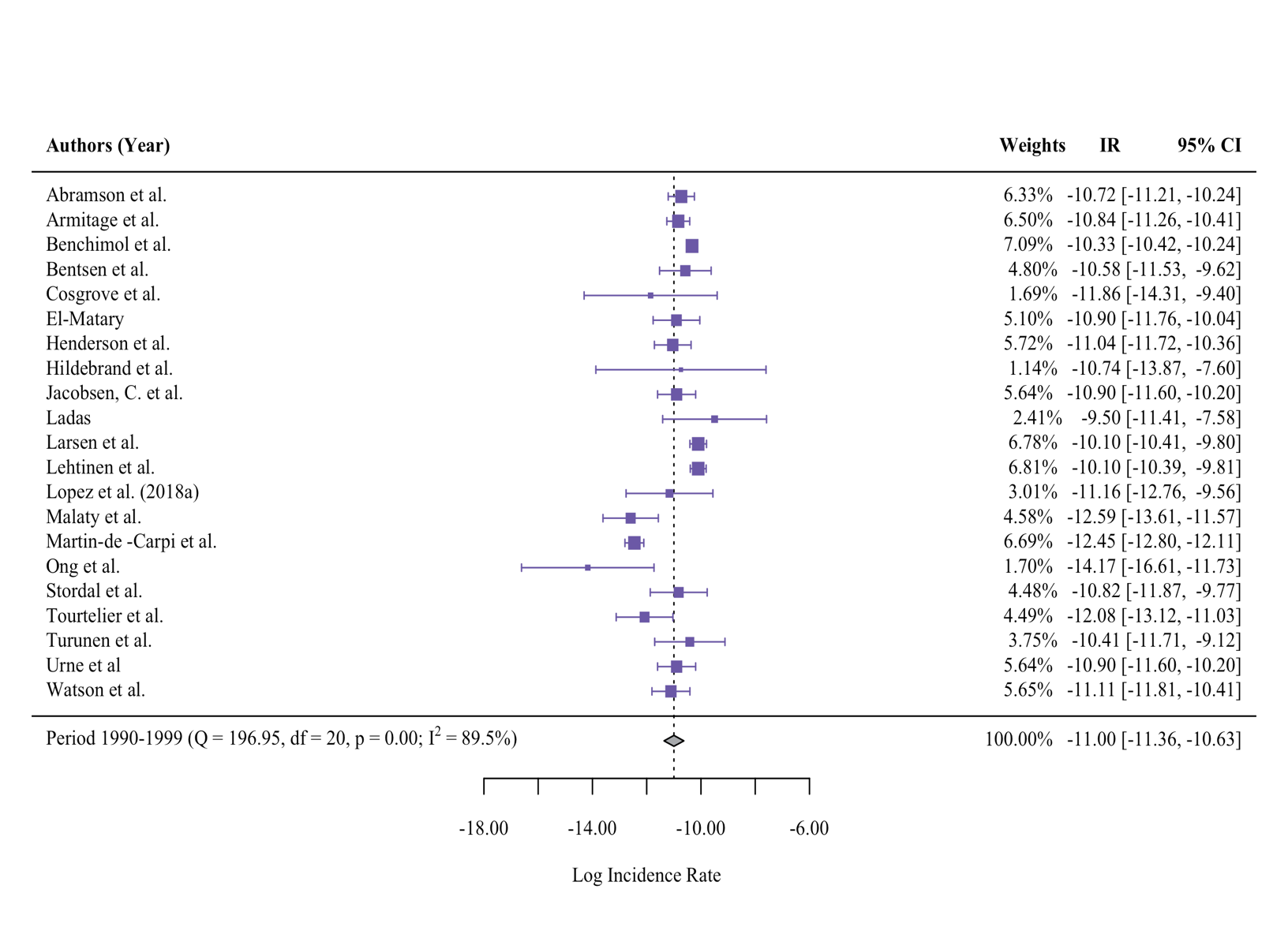


**Fig.S 6:** Forest Plot Studies period 1990-1999


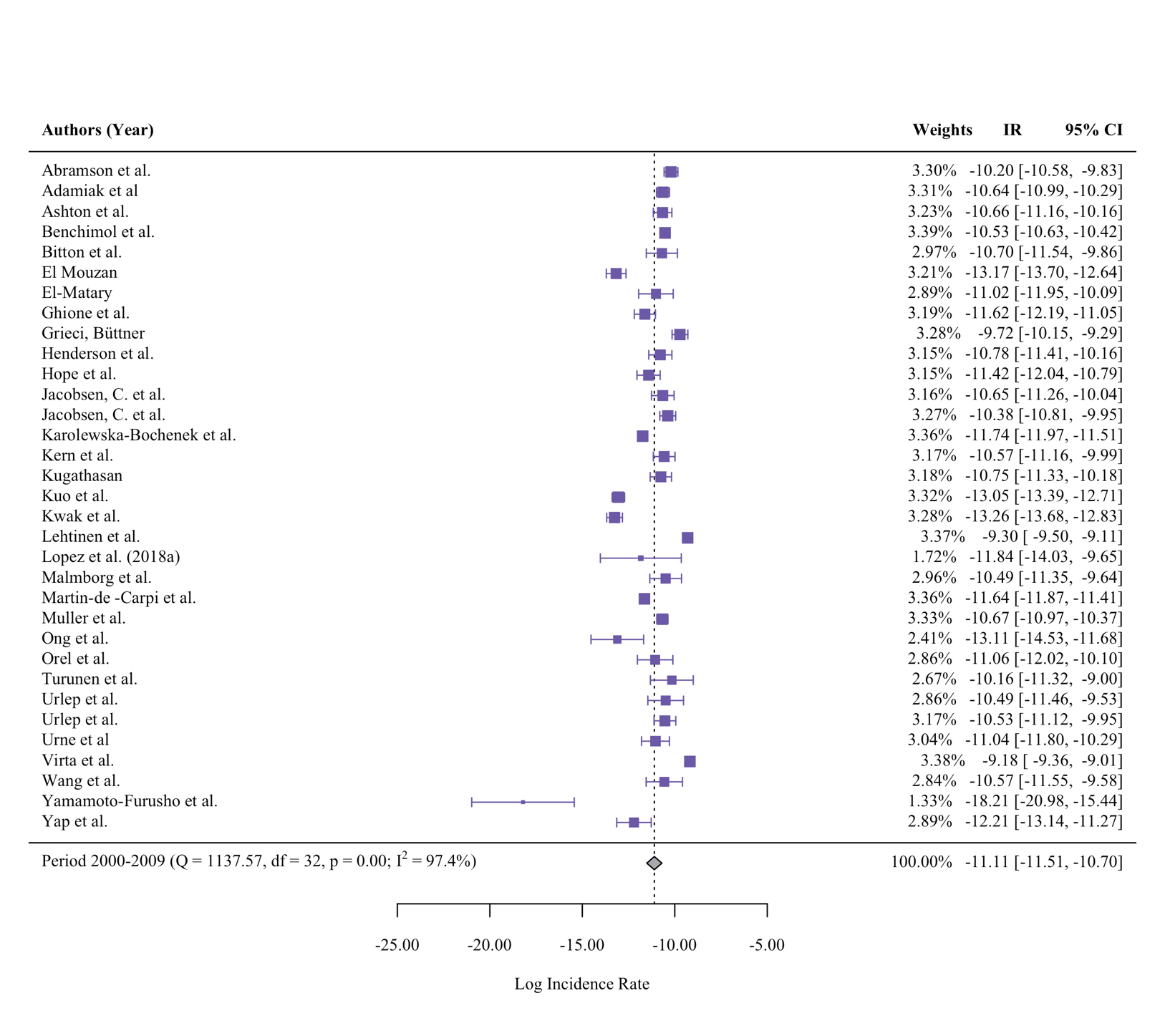


**Fig.S 7:** Forest Plot Studies period 2010-2019


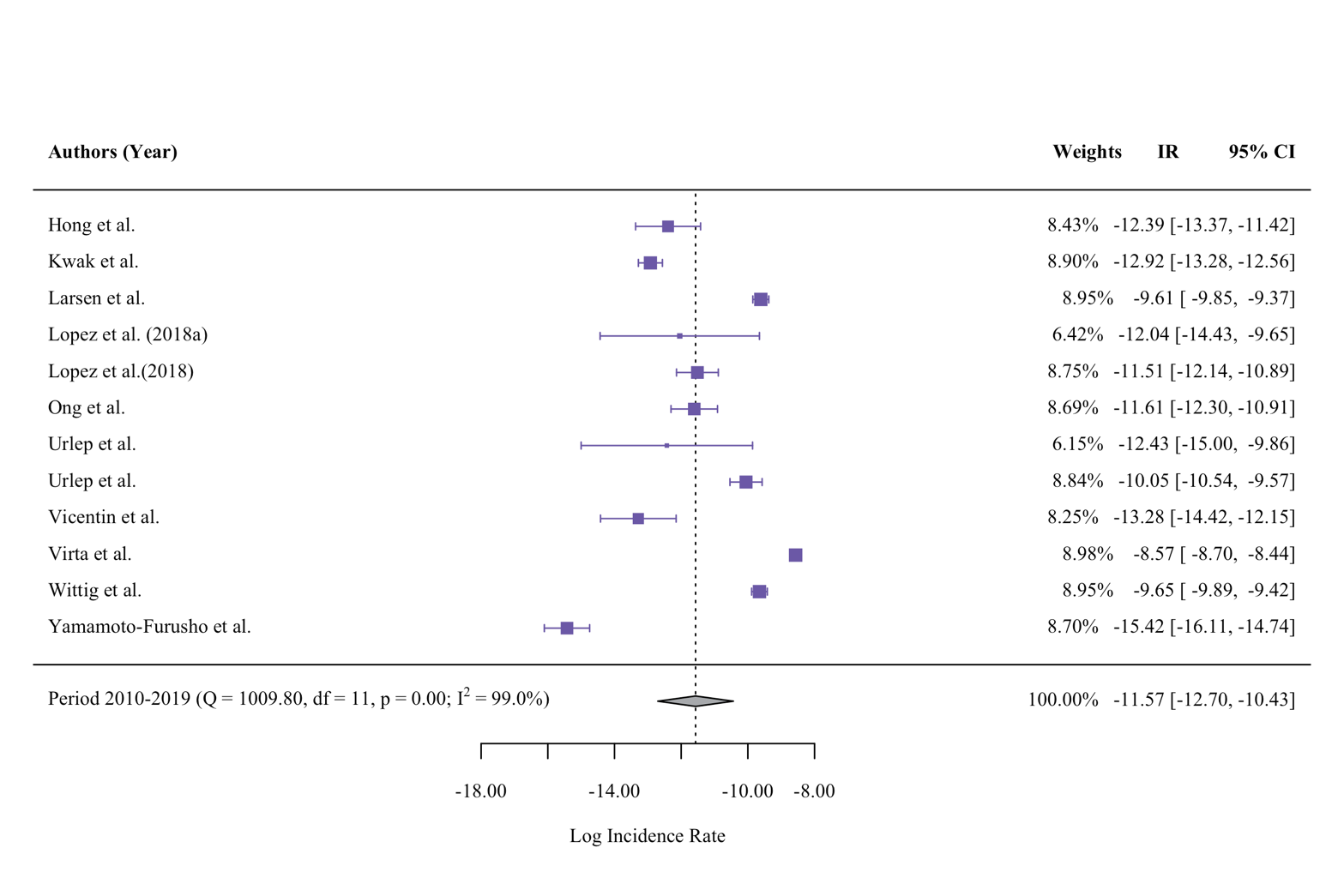


**Fig.S 8:** Forest Plot Studies period 2010-2019

# Supplement Subgroup analysis of the continents

#
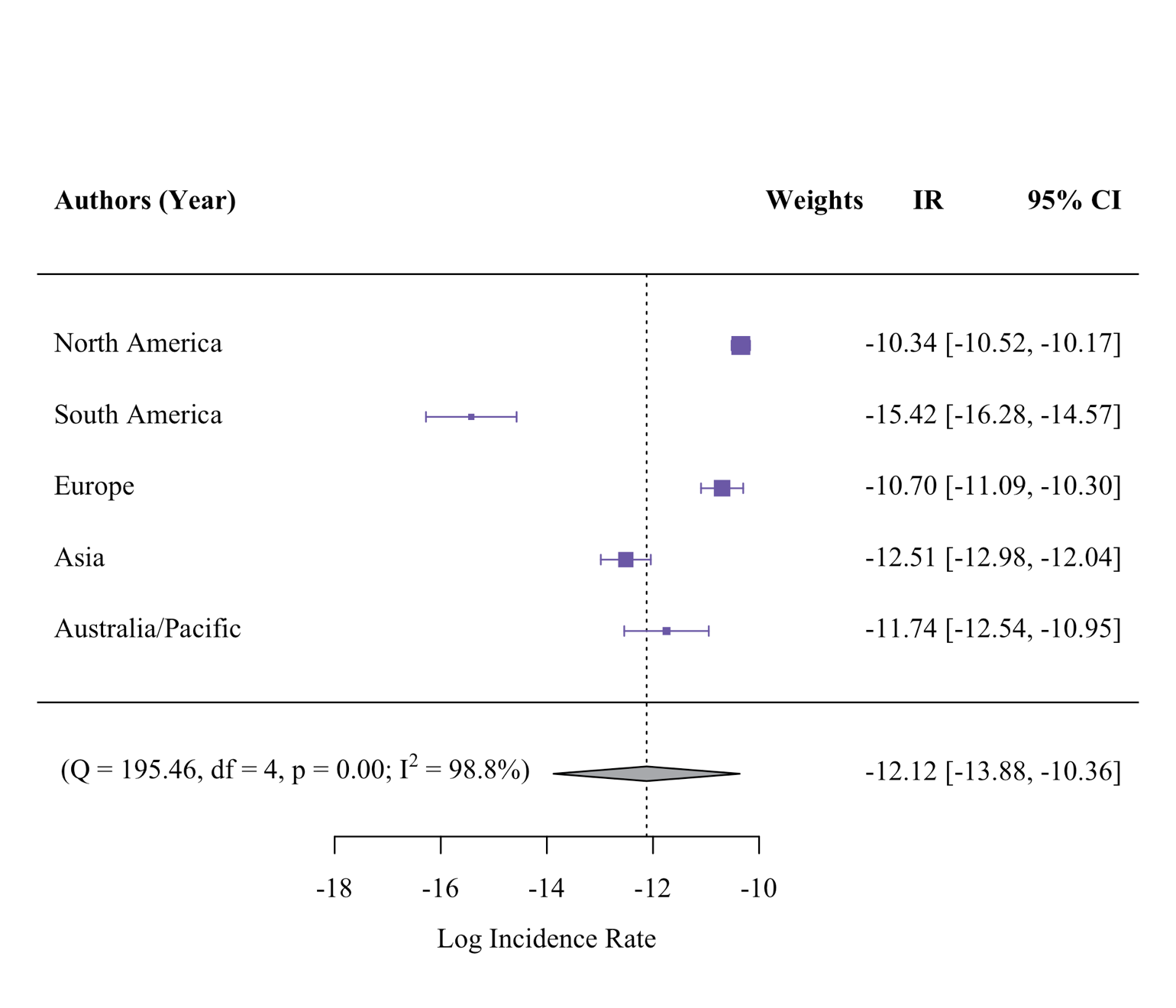


**Fig.S 9:** Forest plot pooled incidence rates by continent

#
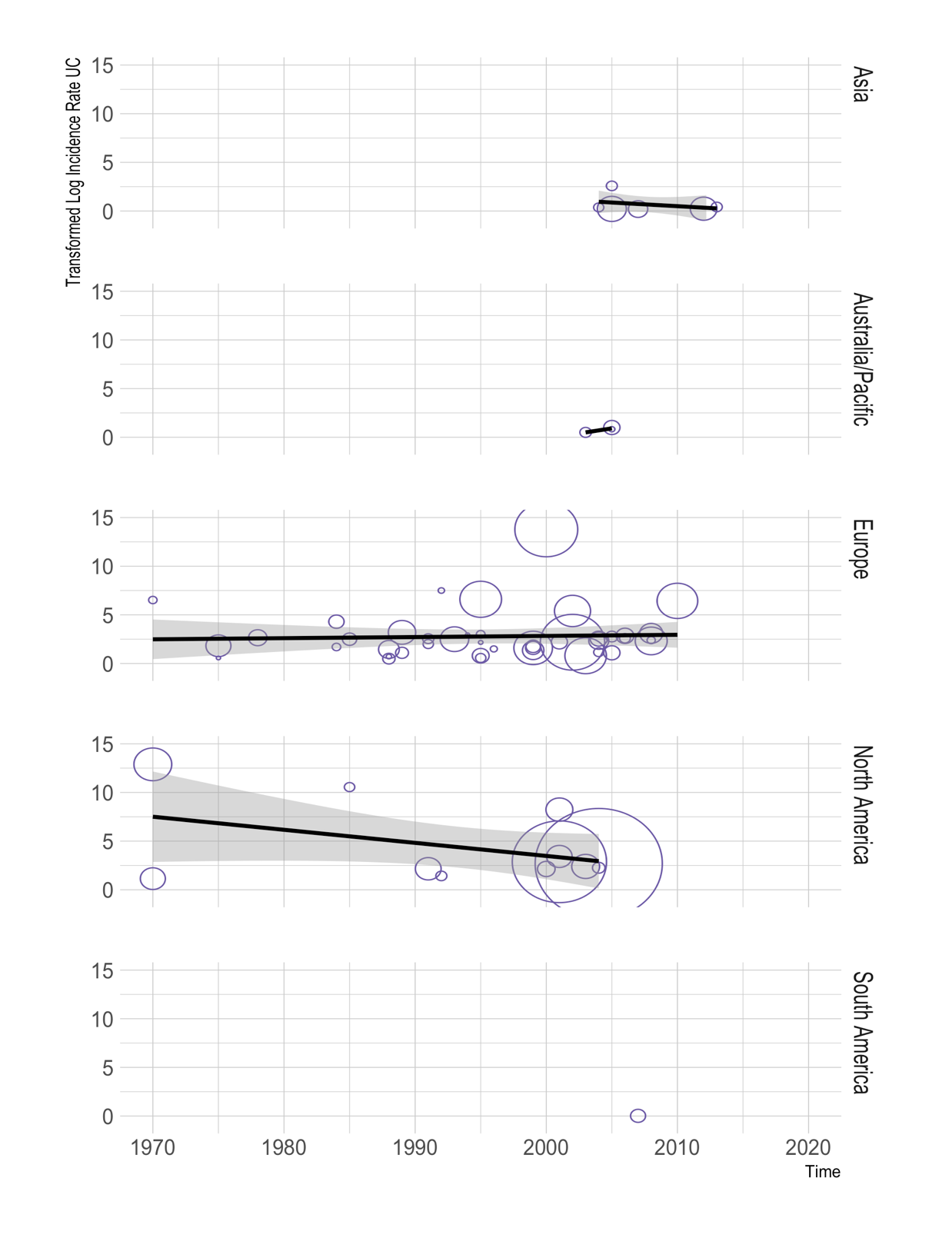


**Fig.S 10:** Meta-regression per continent
